# Lacustrine Shale Oil Enrichment and Mobility in the Lower Cretaceous Shahai Formation, Fuxin Basin (NE China): Integrated Inorganic–Organic Geochemical and Petrographic Evidence

**DOI:** 10.1021/acsomega.5c09312

**Published:** 2025-11-03

**Authors:** Fei Xiao, Hongwei Zhao, Xiaoyong Gao, Dejun Zhang, Yiming Huang, Haihua Zhang, Zhen Zhen, Jianguo Yang, Shichao Li, Yulai Yao, Yujin Zhang

**Affiliations:** a 310457 Shenyang Center of China Geological Survey/Northeast Geological S&T Innovation Center of China Geological Survey, Shenyang, Liaoning 110034, China; b Shale Oil Technology Innovation Center of China Geological Survey, Shenyang, Liaoning 110034, China; c Observation and Research Station of Mesozoic Stratigraphic System in Western Liaoning, MNR, Shenyang, Liaoning 110034, China

## Abstract

The exploration of lacustrine shale oil systems has become a critical frontier in global unconventional energy. Current research primarily focuses on deepwater lacustrine shales in large-sized basins, with limited attention to lacustrine shale oil in oil-coal coexisting strata of small- to medium-sized basins. The Fuxin Basin, a small-sized sedimentary basin in northeast China, is known for its cooccurrence of coal and petroleum resources. The third (K_1_ *sh* ^3^) and fourth (K_1_ *sh* ^4^) members of its Lower Cretaceous Shahai Formation host archetypal coal-measure and lacustrine source rocks, respectively. Therefore, K_1_ *sh* ^4^ is an excellent research object for studying this category of lacustrine shale oil resource. To date, exploration of the Shahai Formation and related geological studies in this basin have focused primarily on conventional clastic reservoirs, coalbed methane, and shale gas, leaving its shale oil potential largely overlooked. Focusing on 23 K_1_ *sh* ^4^ mudstone samples in Well LFD1, this study integrated inorganic–organic geochemical and petrological data to evaluate oil-generation potential, reconstruct paleoclimate and paleodepositional environments, determine the biological origin of organic matter (OM) in these samples, and reveal shale oil enrichment and the controls on oil mobility in the K_1_ *sh* ^4^ lacustrine mudstones. The mudstones are characterized by high OM abundance, mainly Type II kerogen dominated by oil-prone sapropelinite, and midmaturity OM, collectively indicating substantial oil-generation potential. Trace element and molecular geochemical evidence indicates that deposition of these mudstones occurred in a warm, semihumid to semiarid paleoclimate, within a stable, highly restricted, predominantly freshwater (partly brackish) lacustrine environment characterized by weakly oxidizing to weakly reducing, dysoxic conditions. This depositional regime facilitated OM accumulation and preservation, providing a material basis for shale oil enrichment. High Σ*n*C_21–_/Σ*n*C_22+_ and low terrigenous/aquatic ratio (TAR) indicate that OM is predominantly sourced from algal and prokaryotic bacteria, while a low regular sterane/17α-hopane ratio underscores the significant contribution of prokaryotic bacteria. Samples display favorable shale oil enrichment (mainly medium-high oil content), yet oil mobility is restricted by strong oil adsorption onto OM. The oil saturation index is negatively correlated with Σ*n*C_21–_/Σ*n*C_22+_ but positively correlated with TAR. These relationships demonstrate that OM biological origin exerts an intrinsic control on oil mobility: First, although algal/bacterial organisms serve as the primary biological sources, a higher terrigenous contribution (relative to algal/bacterial OM) lowers TOC and adsorbed-oil volumes, thereby increasing the movable oil. Second, at equivalent thermal maturity, low Σ*n*C_21–_/Σ*n*C_22+_ and high TAR values can be associated with larger pore sizes and enhanced reservoir connectivity, facilitating fluid mobility and more complete fractionation of *n*-alkanes within nano- to microscale confinement. This ultimately leads to a more uniform distribution of *n*-alkanes across different carbon numbers in terms of content. This study confirms that the K_1_ *sh* ^4^ lacustrine mudstones in the Fuxin Basin constitute promising targets for future shale oil exploration and provides a pivotal reference for evaluating the lacustrine shale oil potential of the widely distributed Shahai Formation.

## Graphical abstract

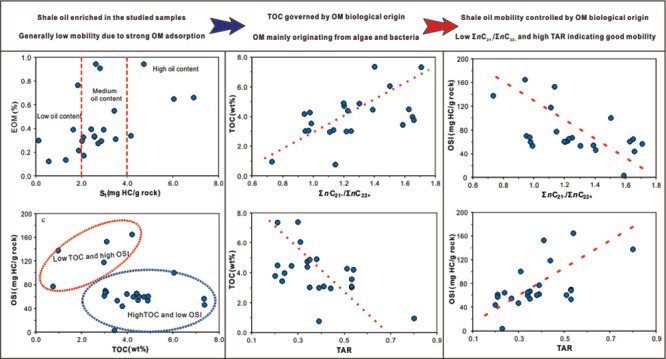

## 1 Introduction

Shale oil refers to petroleum generated within and stored in organic-rich mudstones and shales, or migrated into adjacent, less organic-rich continuous rock intervals.^1,2^ According to the U.S. Energy Information Administration, Russia, the United States, and China possess the world’s three largest technically recoverable shale oil resources.^3^ The shale revolution enabled the United States to achieve commercial shale oil production, thereby realizing energy independence.^4–6^ Inspired by this success, China has intensified shale oil exploration across major petroliferous basins over the past decade. Significant breakthroughs have been made in source rock sequences in the Songliao, Bohai Bay, Ordos, Qaidam, Junggar, and Subei basins,^7–10^ leading to the establishment of four national shale oil demonstration areas: Daqing-Gulong in the Songliao Basin, Shengli-Jiyang in the Bohaibay Basin, Changqing-Longdong in the Ordos Basin, and Xinjiang-Jimusaer in the Junggar Basin.^11^ Unlike the dominant marine shale oil in the United States, China’s shale oil primarily originates from lacustrine deposition, exhibiting strong heterogeneity of mudstones and shales.^8,11,12^ With significant breakthroughs in lacustrine shale oil exploration and development across multiple basins in China, lacustrine shale oil has garnered increasing attention from global oilfield enterprises and research institutions. The enrichment patterns and mobility of shale oil, as key scientific issues constraining its effective development, have become a focal point of research for geologists and geochemists.

Currently, shale oil exploration and research in China have primarily focused on large-sized (>10 × 10^4^ km^2^) lacustrine sedimentary basins, with relatively limited attention paid to medium- (1–10 × 10^4^ km^2^) to small-sized (<1 × 10^4^ km^2^) lacustrine basins. The northeastern region of China is abundant in oil, gas, and coal resources. In addition to large lacustrine sedimentary basins like the Songliao Basin, there are also several medium- and small-sized lacustrine basins containing coal and hydrocarbon deposits.^13^ The Fuxin Basin in western Liaoning Province serves as a representative small-sized basin (Figure 1a,b), in which the Lower Cretaceous Shahai Formation hosts thick coal-measure source rocks in its third member (K_1_ *sh* ^3^) and well-developed lacustrine source rocks in its fourth member (K_1_ *sh* ^4^) (Figure 1c,d).^14,15^ Despite widespread hydrocarbon indications, no commercial oil flow was achieved prior to 2017. During 2016–2017, the Shenyang Center of China Geological Survey drilled two exploratory wells (LFD1 and LFD2). Well LFD2 yielded high-productivity commercial oil flow (15.3 m^3^/day) from the K_1_ *sh* ^4^ gravity-flow conglomerate reservoirs (Figure 1c), marking the basin’s first major hydrocarbon breakthrough.^14,16^ Subsequently, Well SY1, drilled by Team 107 of Northeast Coalfield Geological Bureau and implemented by the Institute of Geology, Chinese Academy of Geological Sciences, also achieved significant discovery in the same formation. This well produced 150–180 m^3^ of oil and 8000–10,000 m^3^ of gas during a 10 h blowout event.^17^ In the field of unconventional oil and gas in the Fuxin Basin, limited coalbed methane production has been implemented in the Liujia and Dongliang areas within the Lower Cretaceous Fuxin and Shahai formations,^18,19^ while shale gas research and exploration primarily focus on the Lower Cretaceous Shahai and Jiufotang formations.^18,20,21^ Nevertheless, shale oil exploration remains largely unexplored, with the corresponding studies being sparse. The oil-source correlation studies indicate that the crude oils in the K_1_ *sh* ^4^ gravity-flow conglomerate reservoir of Well LFD1 and Well LFD2 primarily originate from the K_1_ *sh* ^3^ coal-bearing source rocks.^14,16^ This demonstrates an internally self-sourced petroleum system within the Shahai Formation. Although no evidence of the crude oil generated by the K_1_ *sh* ^4^ lacustrine mudstones has been reported, its proximity (especially its lower part) in depth to that of the K_1_ *sh* ^3^ results in similar thermal maturity. Moreover, the K_1_ *sh* ^4^ lacustrine source rock is likely to exhibit stronger oil-prone characteristics, suggesting the possible potential to generate an industrially significant volume of crude oil and to form effective shale oil accumulation.

**Figure 1:**
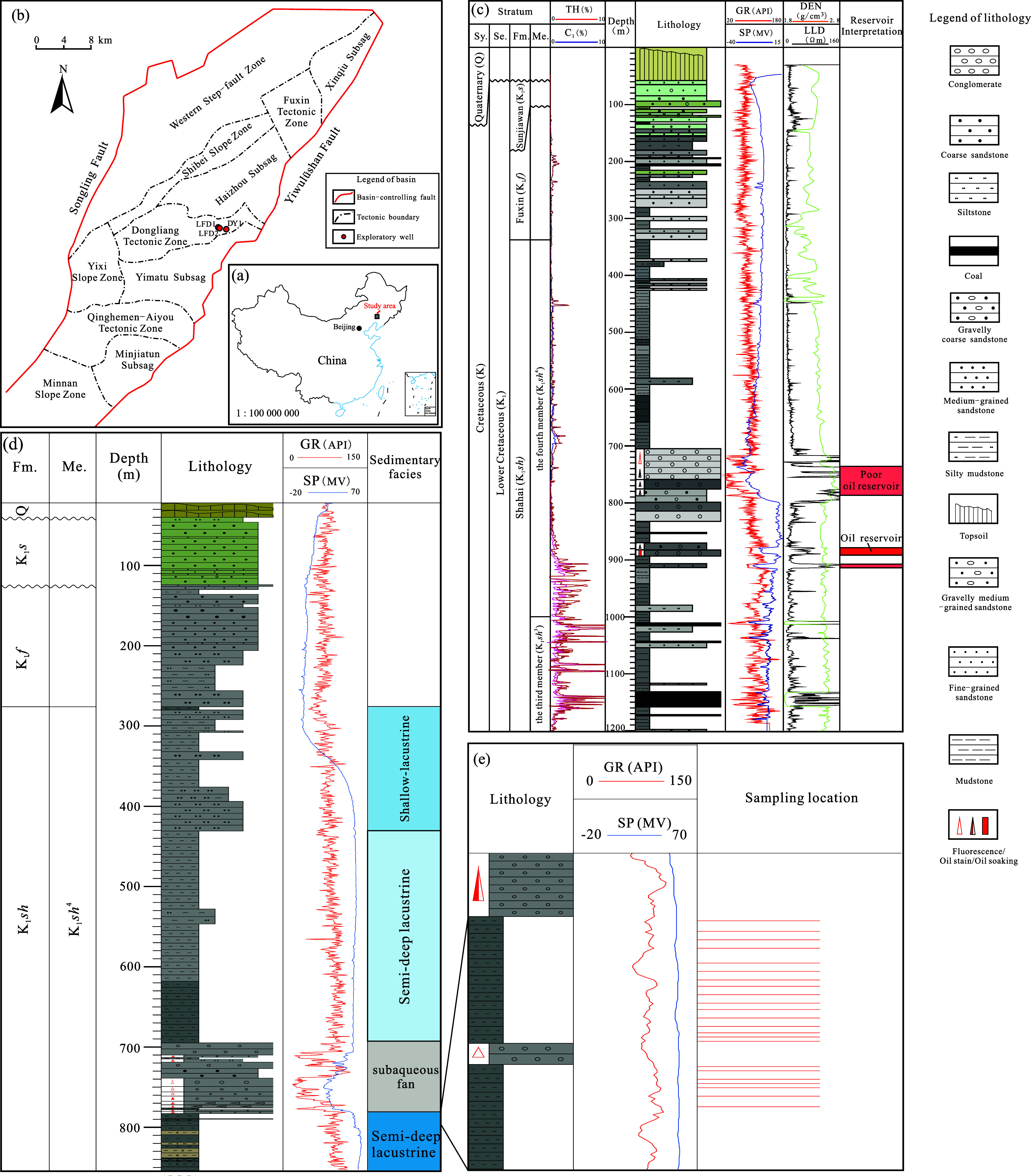
(a) Location of the study area in China (China basemap after China National Bureau of Surveying and Mapping Geographical Information); (b) tectonic units and locations of typical exploratory wells in the Fuxin Basin; (c) composite stratigraphic column of Well LFD2; (d) composite stratigraphic column of Well LFD1; (e) enlarged schematic showing the sampling location. Sy. = system, Se. = series, Fm. = formation, Me. = member, TH = total hydrocarbon, *C*_1_ = percentage of methane (CH_4_), GR = natural gamma-ray logging, SP = spontaneous potential logging, DEN = compensated density logging, and LLD = deep laterolog resistivity logging.

Previous studies have examined the geochemical characteristics of source rocks and oil-source correlations in the Shahai Formation of the Fuxin Basin, as well as the tectonic controls on hydrocarbon accumulation.^14–17,22–25^ Furthermore, reservoir evaluations for shale gas and geological assessments of coalbed methane resources have been performed.^18–21^ However, these works primarily focused on conventional reservoirs and unconventional natural gas with limited attention to shale oil. Using cores from Well LFD1, this study employs an integrated approach combining inorganic geochemistry, organic geochemistry, and organic petrology to analyze the K_1_ *sh* ^4^ lacustrine mudstones. Our objectives are to (1) evaluate the oil-generation potential, (2) reconstruct paleoclimatic and paleodepositional conditions, (3) characterize organic matter (OM) sources, and (4) elucidate shale oil enrichment and mobility in these mudstones.

## 2 Geological Setting

The Fuxin Basin is situated in western Liaoning Province, Northeast China, exhibiting an elongated geometry trending northeast-southwest (Figure 1a,b). It extends approximately 130 km in the north–south direction and 8–20 km in the east–west direction, covering a total area of ∼2000 km^2^.^14^ Tectonically, the basin is located in the eastern segment of the Yanshan Fold Belt at the northeastern margin of the North China Craton. It lies at the intersection of the Tanlu Fault Zone and Chifeng-Kaiyuan Fault, forming a dual-fault graben system bounded westward by the Songling Fault and eastward by the Yiwulüshan Fault (Figure 1b).^18^ The basin infill displays a depression-slope-uplift structural architecture, comprising 11 tectonic units, including the Western Step-fault Zone, the Xinqiu Subsag, the Fuxin Tectonic Zone, the Haizhou Subsag, the Shibei Slope Zone, the Dongliang Tectonic Zone, the Yimatu Subsag, the Yixi Slope Zone, the Qinghemen-Aiyou Tectonic Zone, the Minjiatun Subsag, and the Minnan Slope Zone (Figure 1b). Basement rocks consist of Archean metamorphics and Mesoproterozoic to Neoproterozoic low-grade meta-sandstones and carbonates.^25^ The sedimentary succession comprises the Lower Cretaceous Yixian (K_1_ *y*), Jiufotang (K_1_ *jf*), Shahai (K_1_ *sh*), Fuxin (K_1_ *f*), and Sunjiawan (K_1_ *s*) formations in ascending stratigraphic order (as partly shown in Figure 1c,d). A significant hiatus exists, marked by the absence of the Upper Cretaceous, Paleogene, and Neogene strata.

The source rocks in the Fuxin Basin are predominantly developed in the Jiufotang and Shahai formations, with the Jiufotang Formation’s source rocks primarily consisting of lacustrine deposits, while the Shahai Formation’s source rocks comprise both lacustrine and coal-bearing sediments.^23,25^ From bottom to top, the Shahai Formation is divided into four members defined by sedimentary facies and lithology. The first member (K_1_ *sh* ^1^) comprises red conglomerates deposited in an alluvial-fan setting. The second member (K_1_ *sh* ^2^) consists of yellowish-green conglomerate–sandstone successions that record a second alluvial-fan cycle. The third member (K_1_ *sh* ^3^) is dominated by shore-shallow lacustrine, fluvial, and fan-delta facies, characterized by sandstone and coal-bearing strata hosting well-developed coal-measure source rocks (Figure 1c). The fourth member (K_1_ *sh* ^4^) is marked by thick, dark, deep- to semideep lacustrine mudstones locally interbedded with gravity-flow deposits and hosts thick lacustrine source rocks (Figure 1c and d).^15,20,23,26–28^

The adjacent Well LFD1 and Well LFD2 are situated within the Dongliang Tectonic Zone (Figure 1b), targeting the conventional hydrocarbon potential in the Shahai Formation. Among them, Well LFD1 was completed to a depth of 855 m, encountering Q, K_1_ *s*, K_1_ *f*, and K_1_ *sh* ^4^ (not fully penetrated) (Figure 1d); Well LFD2 was completed to a depth of 1200 m, encountering Q, K_1_ *s*, K_1_ *f*, K_1_ *sh* ^4^, and K_1_ *sh* ^3^ (not fully penetrated) (Figure 1c). The drilling lithology data reveal that K_1_ *sh* ^4^ is dominated by thick layers of dark gray and gray mudstone, interbedded locally with gray sandstone and siltstone, as well as gray and dark gray conglomerate. The K_1_ *sh* ^3^ consists of coal seams, dark gray mudstone, gray sandstone and siltstone, and dark gray conglomerate-bearing coarse sandstone (Figure 1c and d). Coring was conducted in selected intervals of K_1_ *sh* ^4^ in both wells. For the Well LFD1, the coring depth ranges from 763 to 792.18 m (K_1_ *sh* ^4^). The interval of 763–783 m belongs to a subaqueous fan facies with coarse-grained sedimentation, primarily composed of gray oil-stained conglomerate and gray oil-stained breccia. The interval of 783–792.18 m corresponds to a semideep lacustrine depositional environment, with lithology mainly consisting of dark gray mudstone (Figure 1d). Well LFD2 encountered several oil-bearing rock intervals in the lower part of K_1_ *sh* ^4^, with core analysis showing 6.55 m of oil-soaked intervals and 3.81 m of oil-stained intervals. Additionally, wireline log interpretation confirmed a 7.4 m-thick oil pay zone (Figure 1c). Drill-stem testing (DST) and swabbing in the open-hole section (718–930 m) yielded a commercial flow rate of 15.3 m^3^/day oil, representing a major exploration breakthrough in the basin.^24^ Oil-source correlation results reveal that the crude oils originate from the K_1_ *sh* ^3^ coal-measure source rocks,^14,16^ exhibiting characteristics consistent with a self-sourced petroleum system.

## 3 Samples and Methods

Given the close proximity (<0.5 km) of Well LFD1 and Well LFD2, and considering the more systematic coring of the K_1_ *sh* ^4^ in Well LFD1, this study prioritizes 23 source rock samples from this well for comprehensive analysis (Figure 1e). All samples underwent total organic carbon (TOC) quantification, rock pyrolysis, kerogen elemental analysis (C, H, O, S, N), kerogen carbon isotope determination, trace element geochemistry, extraction of soluble OM using the Soxhlet method, column chromatographic fractionation, and gas chromatography (GC) testing. Additionally, six samples were selected for further analyses, including vitrinite reflectance (Ro) measurements, organic maceral analysis, and gas chromatography–mass spectrometry (GC-MS) testing.

### 3.1 TOC Quantification and Rock Pyrolysis

Collected source rock samples were first pulverized and then sieved through a 200-mesh sieve. The sieved material was then dried at a low temperature (<40 °C) prior to subsequent geochemical analyses. Approximately 80–120 mg of powdered samples were accurately weighed into crucibles. To remove inorganic carbon, a hydrochloric acid solution (HCl:deionized water = 1:7 v/v) was added to each sample. After the complete reaction, residual chloride ions were eliminated by repeated rinsing with deionized water and low-temperature drying. Final TOC measurements were conducted using a LECO CS-230 carbon/sulfur analyzer calibrated with certified reference materials.

Rock pyrolysis was conducted by using an OGE-VI Hydrocarbon Analyzer. Sample aliquots of 80–120 mg were weighed and loaded for each analysis. Initially, the temperature was swiftly elevated to 300 °C and maintained at this level for 3 min to quantify the free hydrocarbons (S_1_). Subsequently, the temperature was increased to 600 °C at a rate of 50 °C/min and held constant for 1 min to determine the content of pyrolyzed hydrocarbons (S_2_). The temperature corresponding to the maximum hydrocarbon generation during S_2_ peak evolution (*T*_max_) was recorded.

### 3.2 Organic Petrology Testing

To observe the organic macerals in mudstones, small rock fragments were impregnated with epoxy resin to prepare polished sections. These blocks were sequentially ground on abrasive papers until optimal surface exposure was achieved, followed by final polishing to obtain optical-grade surfaces for petrographic examination. Maceral identification was conducted using a Leica DMRXP microscope equipped with a 50× oil-immersion objective lens under reflected white light and blue-light fluorescence modes.

For the Ro test, samples were crushed to a grain size of 1.00 ± 0.05 mm. Representative samples were mixed with epoxy resin and allowed to cure. Standard rock thin sections were prepared through a three-step sample preparation process that included coarse grinding, fine grinding, and polishing. After the preparation was complete, the thin section surfaces were treated with immersion oil to enhance the optical clarity. Vitrinite measurements were conducted by using a Leica DMRXP microscope equipped with a photomicrophotometer system. Vitrinite particles meeting strict criteria were selected at 50× magnification: (i) Sharp boundaries, (ii) Scratch-free surfaces, and (iii) Homogeneous internal structure. A minimum of 30 measurements per sample was obtained, where the number of vitrinite particles permitted. Mean Ro values were calculated as the final results.

### 3.3 Analysis of Organic Elements and Carbon Isotopes in Kerogen

Initially, kerogen was isolated from the source rock samples. Rock samples were pulverized to pass through a 200-mesh sieve prior to analysis. Subsequently, the samples were treated with HCl and hydrofluoric acid (HF) to remove the inorganic minerals. After demineralization, the samples were extracted by using a Soxhlet extractor with a mixture of dichloromethane and methanol (9:1 v/v) to remove soluble OM. This procedure yielded purified kerogen from core specimens.

Kerogen elemental composition was determined by using a Vario Micro Cube elemental analyzer. During the analysis, carbon (C) and hydrogen (H) in kerogen were oxidized to carbon dioxide (CO_2_) and water (H_2_O), respectively, and their contents were subsequently determined. Concurrently, nitrogen (N), oxygen (O), and sulfur (S) in kerogen were transformed to nitrogen gas (N_2_), carbon monoxide (CO), and sulfur dioxide (SO_2_) under high-temperature conditions, enabling the quantification of these elements.

Kerogen carbon isotope data of mudstone samples (δ^13^C_kerogen_) were acquired by using an IsoPrime EUO E3000 isotope ratio mass spectrometer (GV Instruments) calibrated against the Vienna Pee Dee Belemnite (PDB) standard. Results are reported in per mil (‰) notation with analytical precision better than ± 0.1‰.

### 3.4 Trace Element Analysis

Appropriate quantities of powdered samples were precisely weighed and treated with nitric acid (HNO_3_) and HF (1:1 v/v). The samples were then placed in a heating apparatus, where the temperature was raised to 190 °C and held constant for 24 h to ensure thorough digestion. Once the samples had cooled to ambient temperature, they were reheated to evaporate any residual reagents. Following this, 2 mL of HNO_3_ was added to each sample, which was then evaporated to dryness. Subsequently, 1 mL of HNO_3_ was added for sealing, and the samples were heated in an oven at 130 °C for 3 h. After cooling, the samples were transferred to suitable containers and diluted to a fixed volume. Ultimately, the collected geological samples underwent rigorous analysis through an advanced ELEMENT XR inductively coupled plasma mass spectrometry (ICP-MS) system, which enabled the acquisition of accurate and reliable trace element concentration data.

### 3.5 Soluble OM Extraction, Fractionation, GC, and GC-MS Analysis

Prior to conducting GC and GC-MS analyses, the source rock samples were processed through a series of preliminary steps. Initially, the samples were crushed to pass through a 100-mesh sieve. Approximately 50 g of the crushed samples was then subjected to Soxhlet extraction using dichloromethane as the solvent for 72 h to extract the soluble OM. The asphaltene fraction was subsequently precipitated from the soluble OM using *n*-hexane. Following this, the asphaltene-free soluble OM was separated into saturated hydrocarbons, aromatic hydrocarbons, and nonhydrocarbon fractions through silica gel/alumina column chromatography.

GC was employed to analyze the saturated hydrocarbon fractions of the extracts, yielding compositional data on *n*-alkanes and isoprenoid alkanes. Utilizing an Agilent 7890 gas chromatograph fitted with an HP-5 capillary column (25 m × 0.20 mm × 0.33 μm), the procedure adhered to the following conditions: an initial hold at 60 °C for 2 min, followed by a temperature ramp to 310 °C at 6 °C/min, and a final hold at 310 °C for 20 min. Detection was achieved via a flame ionization detector (FID) with the injector temperature maintained at 300 °C. High-purity helium served as the carrier gas, flowing at a controlled rate of 1.0 mL/min.

GC-MS was employed to analyze saturated and aromatic hydrocarbons, yielding compositional data on terpanes, steranes, and aromatic hydrocarbons. The analysis was performed by using an Agilent 6890N GC/5975 MSD system. For the analysis of saturated hydrocarbons, an HP-5MS capillary column (30 m × 0.25 mm × 0.25 μm) was employed. The injector temperature was set at 300 °C, and the injection was performed in pulsed splitless mode with a constant carrier gas flow of 1.0 mL/min. The temperature program involved an initial hold at 50 °C for 1 min, followed by an increase to 100 °C at a rate of 20 °C/min, and then a further rise to 315 °C at 3 °C/min, where it was held for 16 min. Mass spectrometry was conducted using an EI source at 70 eV, with full scan detection over the range 50–550 amu. The ion source and quadrupole temperatures were maintained at 230 and 150 °C, respectively. For the analysis of aromatic hydrocarbons, a 60 m HP-5MS capillary column was employed. The injector temperature was set to 290 °C. The temperature program started at 50 °C, held for 1 min, and then increased to 310 °C at a rate of 3 °C/min, where it was held for 21 min. The mass spectrometry parameters were consistent with those used for saturated hydrocarbon analysis. Data were processed using MSD ChemStation software with system parameters set to meet the requirements for the separation and identification of hydrocarbon compounds.

## 4 Results

### 4.1 TOC and Rock Pyrolysis Data

Table 1 presents the TOC and rock pyrolysis data for the K_1_ *sh* ^4^ source rock samples from the Well LFD1. The rock pyrolysis data include S_1_, S_2_, and S_3_, hydrogen index (HI, S_2_/TOC), oxygen index (OI, S_3_/TOC), and *T*_max_. Notably, the *T*_max_ value for the sample at a depth of 787.4 m is anomalously high at 541 °C, while the *T*_max_ values for the other samples are relatively consistent, ranging from 433 to 438 °C, with an average of 443 °C. The elevated *T*_max_ value at 787.4 m may be attributed to the diabase intrusion experienced by the sample.^25^

**Table 1:** Geochemical Parameters of Total Organic Carbon (TOC) and Rock Pyrolysis in the K_1_ *sh* ^4^ Source Rock Samples from Well LFD1, Fuxin Basin

sample No.	depth (m)	TOC (wt%)	S_1_ (mg HC/g rock)	S_2_ (mg HC/g rock)	S_3_ (mg CO_2_/g TOC)	S_1_ + S_2_ (mg HC/g rock)	*T*_max_ (°C)	HI (mg HC/g TOC)	OI (mg CO_2_/g TOC)	OSI (mg HC/g rock)
1	783.2	0.75	0.58	1.97	2.23	2.55	444	262.67	297.33	77.33
2	783.7	4.45	2.42	16.36	2.06	18.78	448	367.64	46.29	54.38
3	784.1	3.98	2.56	20.72	2.96	23.28	444	520.60	74.37	64.32
4	784.5	3.09	4.72	12.55	2.61	17.27	433	406.15	84.47	152.75
5	785.2	4.49	2.73	27.44	2.25	30.17	442	611.14	50.11	60.80
6	785.6	3.02	2.11	15.24	2.20	17.35	444	504.64	72.85	69.87
7	786.0	3.07	2.09	17.24	2.08	19.33	442	561.56	67.75	68.08
8	786.3	4.73	2.82	30.12	2.38	32.94	444	636.79	50.32	59.62
9	786.7	4.88	2.62	26.96	2.74	29.58	442	552.46	56.15	53.69
10	787.1	2.99	1.84	14.54	2.76	16.38	445	486.29	92.31	61.54
11	787.4	3.43	0.12	2.08	4.60	2.20	541	60.64	134.11	3.50
12	787.8	4.19	6.91	16.65	2.59	23.56	443	397.37	61.81	164.92
13	788.2	2.96	3.5	14.47	2.93	17.97	444	488.85	98.99	118.24
14	788.5	6.04	6.04	32.85	3.25	38.89	434	543.87	53.81	100.00
15	788.7	0.96	1.32	3.00	2.61	4.32	439	312.50	271.88	137.50
16	788.9	3.03	2.03	11.58	3.28	13.61	445	382.18	108.25	67.00
17	790.1	4.39	2.87	24.91	2.86	27.78	446	567.43	65.15	65.38
18	790.3	3.55	1.88	18.55	2.80	20.43	445	522.54	78.87	52.96
19	790.7	7.37	3.43	46.57	2.34	50.00	444	631.89	31.75	46.54
20	790.9	4.26	2.56	20.93	2.38	23.49	446	491.31	55.87	60.09
21	791.1	3.77	1.64	17.38	3.03	19.02	444	461.01	80.37	43.50
22	791.5	7.34	4.17	46.73	2.52	50.90	439	636.65	34.33	56.81
23	792.0	4.9	2.97	34.08	2.94	37.05	447	695.51	60.00	60.61

### 4.2 Organic Petrology

Microscopic examination of the lacutrine mudstone samples reveals a significant presence of brown, amorphous sapropelinite (sapropel component) under transmitted light, characterized by its dispersed and flocculent appearance. The samples also contain a notable amount of black, blocky inertinite and brown, blocky, and strip-like vitrinite (Figure 2a-I–f-I). Liptinite is absent. Under reflected light with blue-light excitation, nearly all organic components exhibit nonfluorescence (Figure 2a-II–f-II). Quantitative analysis shows that the sapropelinite content ranges from 62 to 67%, averaging 65%. Vitrinite content spans from 10 to 20%, with an average of 16%, while inertinite content varies from 14 to 25%, averaging 19% (Table 2).

**Figure 2:**
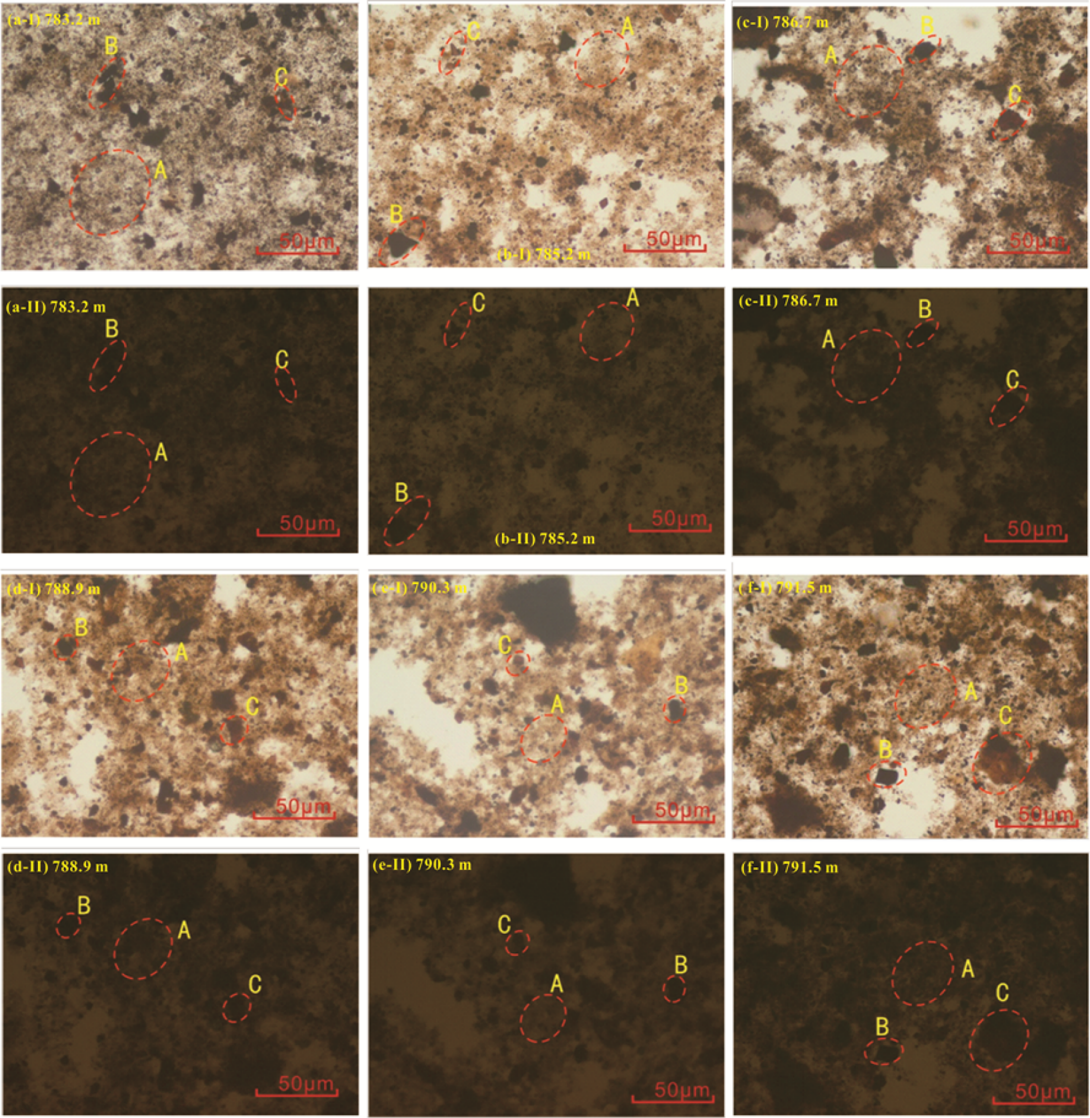
Microscopic characteristics of macerals in the typical K_1_ *sh* ^4^ source rock samples from Well LFD1, Fuxin Basin, under transmitted light (a-I–f-I) and reflected light (a-II–f-II). A = sapropelinite, B = inertinite, and C = vitrinite.

**Table 2:** Maceral Composition and Vitrinite Reflectance Data in the K_1_ *sh* ^4^ Source Rock Samples from Well LFD1, Fuxin Basin^a^

		percentage content of maceral groups (%)				vitrinite reflectance			
sample no.	depth (m)	sapropelinite	liptinite	vitrinite	inertinite	minimum (%)	maximum (%)	mean (%)	number of measurement points
1	783.2	66	0	14	20	0.46	0.78	0.69	16
						0.86	1.33	1.11	23
5	785.2	62	0	14	24	0.62	1.11	0.90	43
9	786.7	65	0	10	25	0.61	1.20	0.94	41
15	788.7	66	0	20	14	0.71	1.18	0.93	47
19	790.7	67	0	19	14	0.68	1.13	0.86	46
22	791.5	64	0	20	16	0.68	1.10	0.87	49

a The sample at the depth of 783.2 m exhibits two distinct groups of vitrinite reflectance values: low-range and high-range measurements.

Ro measurements for the sample at a depth of 783.2 m reveal two distinct sets of values: a higher set averaging 1.11% and a lower set averaging 0.69% (Table 2). Research has previously documented thermal baking in certain areas of the Fuxin Basin due to diabase intrusion.^25^ It is thus inferred that the anomalous Ro values can be due to abnormal thermal effects. The relatively brief heating period likely prevented the samples from reaching thermal equilibrium values, thereby producing this phenomenon. For the remaining samples, Ro values range from 0.86 to 0.94%, with an average of 0.90% (Table 2).

### 4.3 Organic Elements and Carbon Isotopes of Kerogen

Table 3 presents the organic elemental and carbon isotope data for kerogen in the K_1_ *sh* ^4^ source rocks. The organic elements of kerogen are composed of C, H, O, S, and N. C exhibits the highest percentage content, varying from 30.33 to 39.21%, averaging 34.96%. O is the second most abundant element, with a percentage content ranging from 4.66 to 12.56%, averaging 10.19%. S content spans from 1.95 to 8.37%, averaging 4.95%, while H content ranges from 1.95 to 5.42%, averaging 4.18%. N has the lowest percentage content, ranging from 0.52 to 1.11%, averaging 0.81%. The H/C atomic ratio of kerogen organic elements ranges from 0.73 to 1.69, averaging 1.43, and the O/C atomic ratio ranges from 0.09 to 0.27, averaging 0.22. Kerogen carbon isotope values (δ^13^C_kerogen_, PDB) range from −27.00 to −25.60‰, averaging −26.29‰.

**Table 3:** Organic Elemental Composition and Carbon Isotopic Data of Kerogen, Extractable OM Content, and Compositional Groups in the K_1_ *sh* ^4^ Source Rock Samples from Well LFD1, Fuxin Basin^a^

											percentage content of group components (%)				
sample no.	depth (m)	C (%)	H (%)	O (%)	S (%)	N (%)	H/C	O/C	δ^13^C_kerogen_ (‰, PDB)	EOM (%)	SAT	ARO	NON	ASP	(SAT + ARO)/ASP
1	783.2	34.24	3.12	12.23	2.57	0.66	1.10	0.27	–25.60	1.23	45.17	22.98	20.65	11.20	6.09
2	783.7	33.18	2.12	11.77	5.02	0.94	0.77	0.27	–26.10	3.93	37.31	21.87	22.45	18.37	3.22
3	784.1	34.82	4.17	9.42	1.95	0.89	1.44	0.20	–26.60	3.32	34.29	21.13	32.07	12.51	4.43
4	784.5	37.94	4.03	4.66	6.53	0.75	1.27	0.09	–26.50	9.44	39.57	22.99	28.20	9.25	6.77
5	785.2	32.47	3.66	9.59	5.17	0.83	1.35	0.22	–26.50	2.73	31.28	19.49	27.52	21.70	2.34
6	785.6	33.15	4.33	10.84	6.69	0.93	1.57	0.25	–26.80	1.70	34.79	21.88	27.50	15.83	3.58
7	786.0	31.06	3.19	8.19	8.37	0.85	1.23	0.20	–26.40	3.25	32.82	21.50	29.70	15.98	3.40
8	786.3	32.27	1.95	9.92	5.66	0.73	0.73	0.23	–26.60	9.08	35.49	22.10	33.23	9.19	6.27
9	786.7	37.84	4.85	11.45	5.19	0.75	1.54	0.23	–26.20	9.44	39.95	20.83	25.49	13.73	4.43
10	787.1	36.10	4.54	11.93	3.29	0.85	1.51	0.25	–26.00	7.65	39.98	19.91	28.25	11.86	5.05
11	787.4	38.78	5.39	12.16	5.06	0.94	1.67	0.24	–26.30	2.97	35.52	21.85	27.18	15.45	3.71
12	787.8	31.52	3.42	10.67	5.02	0.52	1.30	0.25	–25.90	6.59	48.04	19.20	22.80	9.96	6.75
13	788.2	35.08	4.56	8.15	5.94	0.69	1.56	0.17	–26.20	3.09	34.28	25.93	32.86	6.93	8.69
14	788.5	36.84	5.19	9.82	3.35	0.95	1.69	0.20	–26.10	6.50	30.89	26.09	32.53	10.49	5.43
15	788.7	34.66	4.44	12.56	5.80	0.67	1.54	0.27	–26.00	1.37	50.21	22.54	19.59	7.66	9.49
16	788.9	33.32	3.66	11.06	7.63	0.60	1.32	0.25	–26.20	2.95	42.03	21.52	20.52	15.93	3.99
17	790.1	38.95	5.01	11.86	6.69	0.85	1.54	0.23	–26.00	2.96	38.14	23.60	28.44	9.82	6.29
18	790.3	30.33	3.64	9.87	6.45	0.71	1.44	0.24	–26.40	2.14	37.26	23.25	24.84	14.65	4.13
19	790.7	36.15	5.07	8.97	2.65	0.98	1.68	0.19	–26.10	5.49	34.43	23.73	27.02	14.82	3.92
20	790.9	33.99	4.72	11.17	2.48	0.68	1.67	0.25	–26.30	3.33	39.67	24.42	26.52	9.39	6.82
21	791.1	38.90	5.07	10.42	3.02	1.08	1.57	0.20	–27.00	3.90	34.13	22.38	32.30	11.20	5.05
22	791.5	39.21	5.42	9.03	4.11	1.11	1.66	0.17	–26.70	3.39	38.11	22.31	16.35	23.23	2.60
23	792.0	33.35	4.67	8.53	5.31	0.73	1.68	0.19	–26.10	3.90	38.04	24.84	22.33	14.79	4.25

a SAT = saturates; ARO = aromatics; NON = nonhydrocarbons; ASP = asphaltenes.

### 4.4 Trace Element

The test results of the main trace element abundances in the K_1_ *sh* ^4^ source rocks and the relevant geochemical parameters are shown in Table 4. The trace elements used in this study include strontium (Sr), copper (Cu), rubidium (Rb), vanadium­(V), nickel (Ni), uranium (U), thorium (Th), molybdenum (Mo), boron (B), and gallium (Ga). Based on these elements, the paleoclimate parameters Sr/Cu and Rb/Sr, the paleoredox parameters V/(V + Ni), Th/U, the δU index (2U/(U + Th/3)), and Mo/TOC, as well as the paleosalinity parameter B/Ga, were calculated.

**Table 4:** Trace Element Concentrations and Geochemical Parameters in the K_1_ *sh* ^4^ Source Rock Samples from Well LFD1, Fuxin Basin^a^

		trace element concentrations (ppm)										trace element geochemical parameters						
sample no.	depth (m)	Sr	Cu	Rb	V	Ni	U	Th	Mo	B	Ga	Sr/Cu	Rb/Sr	V/(V + Ni)	Th/U	δU	Mo/TOC	B/Ga
1	783.2	93	27	97	67	22	1.8	0.8	0.5	29	18	3.51	1.04	0.75	0.43	1.75	0.72	1.67
2	783.7	34	17	70	39	6	0.5	0.3	0.3	26	1	2.02	2.06	0.86	0.58	1.68	0.07	32.61
3	784.1	166	20	75	59	13	1.1	0.6	0.6	27	7	8.22	0.45	0.82	0.52	1.71	0.15	3.76
4	784.5	368	7	42	18	19	0.2	0.6	0.3	12	3	54.71	0.12	0.49	3.77	0.89	0.11	3.72
5	785.2	193	19	82	45	10	0.8	0.5	0.4	26	16	9.91	0.43	0.82	0.63	1.65	0.10	1.65
6	785.6	181	7	25	28	9	0.4	0.4	0.4	12	4	24.36	0.14	0.75	1.11	1.46	0.13	3.35
7	786.0	160	20	83	62	22	1.3	0.5	0.8	28	12	7.94	0.52	0.74	0.41	1.76	0.26	2.41
8	786.3	101	15	95	51	5	0.6	0.3	0.4	28	10	6.60	0.94	0.92	0.61	1.66	0.08	2.87
9	786.7	160	22	91	54	16	1.3	0.5	0.7	27	13	7.40	0.57	0.77	0.35	1.79	0.15	2.12
10	787.1	197	24	90	55	24	1.2	0.9	0.9	22	8	8.30	0.46	0.69	0.73	1.61	0.31	2.79
11	787.4	209	26	104	60	16	1.3	0.6	0.7	28	19	7.89	0.50	0.78	0.42	1.76	0.22	1.50
12	787.8	130	17	82	48	33	0.3	0.8	1.4	16	4	7.84	0.63	0.59	2.53	1.09	0.33	3.54
13	788.2	38	5	71	52	12	0.7	0.6	0.3	21	3	7.64	1.85	0.82	0.90	1.54	0.11	6.23
14	788.5	471	40	106	55	35	1.3	1.8	0.8	18	9	11.89	0.22	0.61	1.34	1.38	0.14	2.01
15	788.7	65	106	17	58	185	N/A	2.6	12.0	13	15	0.61	0.26	0.24	N/A	N/A	12.51	0.84
16	788.9	86	10	32	34	10	0.0	0.4	0.4	15	4	8.38	0.37	0.77	12.32	0.39	0.12	4.12
17	790.1	284	27	117	59	24	1.4	0.7	0.8	27	30	10.64	0.41	0.71	0.47	1.73	0.18	0.90
18	790.3	133	10	42	33	7	0.1	0.4	0.6	12	4	13.52	0.32	0.82	4.93	0.76	0.17	3.24
19	790.7	220	16	65	39	18	0.5	0.6	0.7	12	5	13.52	0.29	0.69	1.35	1.38	0.09	2.60
20	790.9	332	7	65	33	10	1.2	0.6	0.2	15	6	46.54	0.20	0.77	0.46	1.73	0.05	2.57
21	791.1	374	20	63	28	23	0.5	0.6	0.9	11	7	18.34	0.17	0.55	1.27	1.41	0.24	1.43
22	791.5	368	21	65	46	38	0.7	1.5	0.9	16	9	17.63	0.18	0.55	2.28	1.14	0.13	1.87
23	792.0	254	34	47	62	21	0.9	0.7	0.7	22	5	7.48	0.19	0.75	0.79	1.58	0.14	4.66

a N/A = data are not available.

### 4.5 Extractable OM Content, Compositional Groups, and Molecular Geochemistry

#### 4.5.1 Extractable OM Content and Compositional Groups

In the K_1_ *sh* ^4^ source rocks from Well LFD1, the extractable OM (EOM) content ranges from 1.23% to 9.44%, with an average of 4.36% (Table 3). The compositional groups are ordered as follows: saturated hydrocarbons > nonhydrocarbons > aromatic hydrocarbons > asphaltenes (Table 3). More specifically, the percentage content of saturated hydrocarbons ranges from 30.89 to 50.21%, with an average of 37.89%. Aromatic hydrocarbons account for 19.20–26.09%, averaging 22.45%. Nonhydrocarbons range from 16.36 to 33.23%, averaging 26.45%. Asphaltenes range from 6.93 to 23.23%, with an average of 13.22%. The ratio of the combined percentage contents of saturated and aromatic hydrocarbons to those of asphaltenes ranges from 2.34 to 9.49, averaging 5.07.

#### 4.5.2 Chain Alkanes

The chain alkanes within the EOM of the source rocks are composed of *n*-alkanes and isoprenoid alkanes (Figure 3a). The mass chromatogram at *m*/*z* 85 illustrates that the carbon number of *n*-alkanes predominantly spans from *n*C_13_ to *n*C_32_, displaying a unimodal distribution pattern (Figure 3a). The primary peak carbon is typically *n*C_21_, except for one sample at a depth of 791.5 m, which exhibits *n*C_17_, with no evident carbon number dominance. Isoprenoid alkanes are primarily composed of pristane (Pr) and phytane (Ph), which are present in comparable abundances. Geochemical parameters associated with chain alkanes are detailed in Table 5.

**Figure 3:**
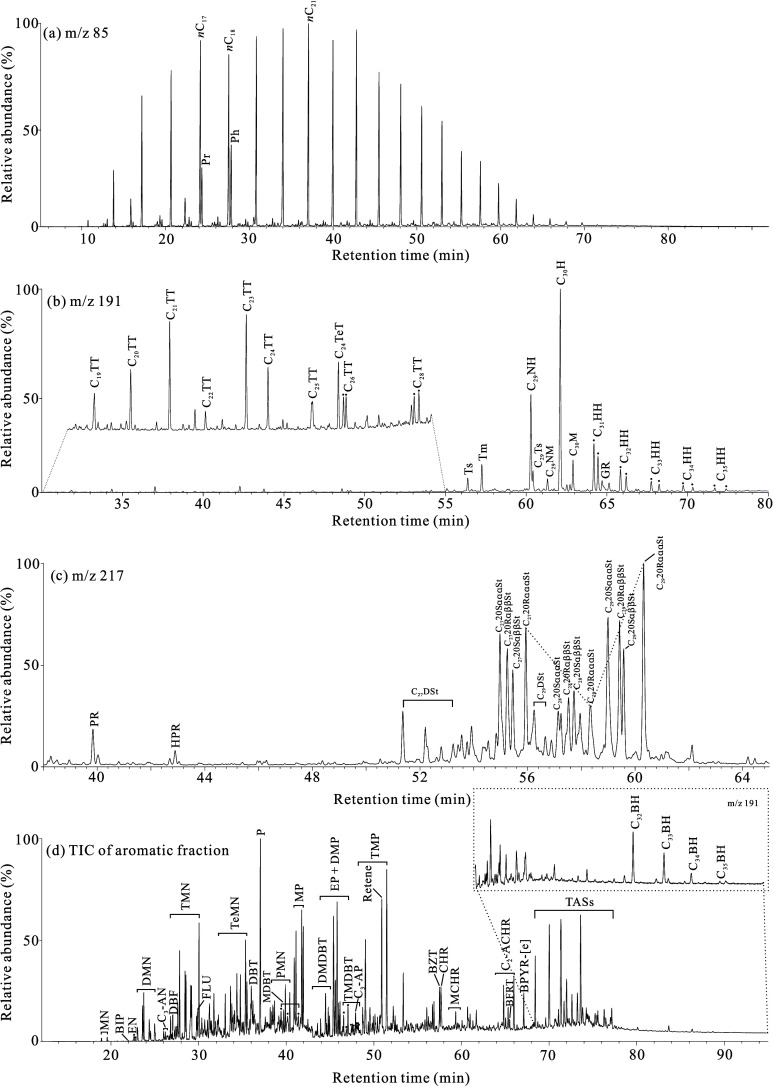
Representative mass chromatograms of saturated hydrocarbons (*m*/*z* 85, 191, 217) and total ion chromatogram (TIC) of aromatic fraction in the K_1_ *sh* ^4^ lacustrine source rock from Well LFD1 (791.5 m), Fuxin Basin, illustrating the characteristic distributions of (a) chain alkanes, (b) terpanes, (c) steranes, and (d) aromatic hydrocarbon compounds. Pr = Pristane, Ph = Phytane, *n*C_*x*_ = normal alkane with x carbon number, TT = tricyclic terpane; TeT = tetracyclic terpane, Ts = C_27_ 22,29,30-trisnorneohopane, Tm = C_27_ 22,29,30-trisnorhopane, NH = norhopane, C_29_Ts = C_29_ 18α­(*H*),21β­(*H*)-30-norneohopane, NM = normoretane, H = hopane, M = moretane, HH = homohopane, GR = gammacerane, PR = pregnane, HPR = homopregnane, DSt = diasterane, St = regular sterane, ααα = 5α­(H), 14α­(H), 17α­(H), αββ = 5α­(H), 14β­(H), 17β­(H), MN = methylnaphthalene, BIP = biphenyl, EN = ethylnaphthalene, DMN = dimethylnaphthalene, C_3_-AN = C_3_ alkylnaphthalene, DBF = dibenzofuran, TMN = trimethylnaphthalene, FLU = fluorene, TeMN = tetramethylnaphthalene, DBT = dibenzothiophene, *P* = phenanthrene, MDBT = methyldibenzothiophene, PMN = pentamethylnaphthalene, MP = methylphenanthrene, DMDBT = dimethyldibenzothiophene, EP = ethylphenanthrene, DMP = dimethylphenanthrene, TMDBT = trimethyldibenzothiophene, C_3_-AP = C_3_ alkylphenanthrene, TMP = trimethylphenanthrene, BZT = benzo­[a]­anthracene, CHR = chrysene, MCHR = methylchrysene, C_3_-ACHR = C_3_ alkylchrysene, BFRT = benzofluoranthene, BPYR-[e] = benzo­[e]­pyrene, TASs = triaromatic steroid series, BH = benzohopane.

**Table 5:** Molecular Geochemical Parameters of Chain Alkanes in the K_1_ *sh* ^4^ Source Rock Samples from Well LFD1, Fuxin Basin^a^

sample	depth (m)	*C*_max_	CPI_12–34_	Σ*n*C_21–_/Σ*n*C_22+_	(*n*C_21_ + *n*C_22_)/(*n*C_28_ + *n*C_29_)	TAR	*P*_aq_	Pr/Ph	Pr/*n*C_17_	Ph/*n*C_18_
1	783.2	*n*C_21_	1.15	1.15	3.76	0.39	0.82	1.08	0.74	0.67
2	783.7	*n*C_21_	1.14	1.39	4.29	0.27	0.85	1.06	0.64	0.61
3	784.1	*n*C_21_	1.15	1.65	3.85	0.24	0.80	0.85	0.56	0.73
4	784.5	*n*C_21_	1.15	1.13	2.95	0.41	0.80	0.82	0.66	0.86
5	785.2	*n*C_21_	1.13	1.63	4.40	0.21	0.85	0.87	0.54	0.70
6	785.6	*n*C_21_	1.14	0.95	2.18	0.53	0.77	0.95	0.58	0.66
7	786.0	*n*C_21_	1.12	0.97	2.23	0.53	0.75	0.83	0.56	0.71
8	786.3	*n*C_21_	1.12	1.20	3.26	0.34	0.82	0.84	0.53	0.69
9	786.7	*n*C_21_	1.14	1.30	2.89	0.35	0.79	0.85	0.58	0.76
10	787.1	*n*C_21_	1.13	1.22	2.78	0.39	0.76	0.82	0.58	0.77
11	787.4	*n*C_21_	1.15	1.59	4.27	0.23	0.85	0.88	0.55	0.70
12	787.8	*n*C_21_	1.14	0.94	2.38	0.54	0.76	0.82	0.67	0.85
13	788.2	*n*C_21_	1.12	1.11	2.50	0.44	0.76	0.84	0.56	0.72
14	788.5	*n*C_21_	1.18	1.50	3.27	0.31	0.78	0.87	0.58	0.74
15	788.7	*n*C_21_	1.12	0.73	2.02	0.80	0.72	0.81	0.74	0.89
16	788.9	*n*C_21_	1.16	1.25	3.45	0.35	0.81	1.04	0.64	0.64
17	790.1	*n*C_21_	1.13	1.23	3.43	0.34	0.80	0.89	0.60	0.72
18	790.3	*n*C_21_	1.14	0.99	2.10	0.53	0.72	0.84	0.57	0.74
19	790.7	*n*C_21_	1.13	1.40	3.14	0.30	0.80	0.84	0.51	0.66
20	790.9	*n*C_21_	1.13	0.98	2.41	0.51	0.76	0.82	0.60	0.77
21	791.1	*n*C_21_	1.13	1.66	5.08	0.20	0.85	0.85	0.57	0.74
22	791.5	*n*C_17_	1.15	1.71	4.23	0.21	0.84	0.84	0.51	0.67
23	792.0	*n*C_21_	1.13	1.20	3.02	0.38	0.78	0.86	0.51	0.64

a *C*_max_ = Main peak carbon number; CPI_12–34_ = 2 × Σodd (*n*C_13_–*n*C_33_)/[Σeven (*n*C_14_–*n*C_34_) + Σeven (*n*C_12_–*n*C_32_)]; TAR = (*n*C_27_ + *n*C_29_ + *n*C_31_)/(*n*C_15_ + *n*C_17_ + *n*C_19_); *P*_aq_ = (*n*C_23_ + *n*C_25_)/(*n*C_23_ + *n*C_25_ + *n*C_29_ + *n*C_31_).

#### 4.5.3 Terpanes and Steranes

The samples contain terpanes, which include tri-, tetra-, and pentacyclic terpanes (Figure 3b). The mass chromatogram at *m*/*z* 191 reveals (Figure 3b) that tri- and tetracyclic terpanes are much less abundant than pentacyclic terpanes. Tricyclic terpanes display a normal distribution with C_23_ as the main peak, while the C_24_ tetracyclic terpane is more abundant than the individual isomer of C_26_ tricyclic terpanes. Pentacyclic terpanes are mainly composed of the hopane series and gammacerane, with C_30_ hopane being the most abundant in the hopane series. The abundance of C_27_ 22,29,30-trisnorneohopane (Ts) is lower than that of C_27_ 22,29,30-trisnorhopane (Tm), and the abundance of C_31_–C_35_ homohopanes decreases stepwise. Gammacerane is present in a very low abundance.

The steranes in the samples are predominantly short-side-chain steranes (pregnenane and homopregnenane), regular steranes, and rearranged steranes (Figure 3c). The mass chromatogram at *m*/*z* 217 illustrates (Figure 3c) that short-side-chain steranes and regular steranes are relatively less abundant, with regular steranes being the most prevalent. The C_27_–C_28_–C_29_ regular steranes show an asymmetric V-shaped distribution, with the lowest abundance being at the C_28_ regular sterane. The abundances of C_27_ and C_29_ regular steranes are not significantly different, with C_29_ regular sterane being more abundant.

Geochemical parameters related to terpanes and steranes are detailed in Table 6.

**Table 6:** Molecular Geochemical Parameters of Terpanes and Steranes in the K_1_ *sh* ^4^ Source Rock Samples from Well LFD1, Fuxin Basin^a^

sample no.	depth (m)	C_31_ 22S/(22S+22R)	C_32_ 22S/(22S+22R)	C_29_ 20S/(20S+20R)	C_29_ ββ/(ββ+αα)	TMNr	TeMNr	F_1_	F_2_	GR/C_30_H	DBTs/DBFs	C_31_HH-22R/C_30_H	C_26_TT/C_25_TT	C_35_HH/C_34_HH	C_29_NH/C_30_H	C_24_TeT/C_26_TT	TTs/H	St/H
1	783.2	0.58	0.57	0.49	0.37	0.24	0.44	0.44	0.25	0.04	0.49	0.18	1.39	0.47	0.45	0.96	0.06	0.16
5	785.2	0.58	0.59	0.48	0.36	0.26	0.46	0.43	0.24	0.06	0.48	0.17	1.25	0.58	0.50	1.01	0.07	0.26
9	786.7	0.58	0.59	0.50	0.38	0.28	0.46	0.44	0.24	0.05	0.41	0.18	1.34	0.55	0.47	0.85	0.06	0.23
15	788.7	0.58	0.57	0.48	0.36	0.27	0.45	0.44	0.24	0.07	0.45	0.17	1.28	0.53	0.50	0.99	0.08	0.28
19	790.7	0.58	0.59	0.48	0.35	0.23	0.43	0.44	0.24	0.07	0.53	0.17	1.26	0.62	0.50	1.03	0.07	0.25
22	791.5	0.58	0.59	0.50	0.38	0.27	0.44	0.44	0.24	0.06	0.47	0.17	1.37	0.61	0.49	0.92	0.08	0.28

a C_31_ 22S/(22S+22R) = C_31_ homohopane 22S/(C_31_ homohopane 22S + C_31_ homohopane 22R); C_32_ 22S/(22S+22R) = C_32_ homohopane 22S/(C_32_ homohopane 22S + C_32_ homohopane 22R); C_29_ 20S/(20S+20R) = C_29_ 5α­(H), 14α­(H), 17α­(H)-sterane 20S/(C_29_ 5α­(H), 14α­(H), 17α­(H)-sterane 20S + C_29_ 5α­(H), 14α­(H), 17α­(H)-sterane 20R); C_29_ ββ/(ββ+αα) = (C_29_ 5α­(H), 14β­(H), 17β­(H)-sterane 20S + C_29_ 5α­(H), 14β­(H), 17β­(H)-sterane 20R)/(C_29_ 5α­(H), 14β­(H), 17β­(H)-sterane 20S + C_29_ 5α­(H), 14β­(H), 17β­(H)-sterane 20R + C_29_ 5α­(H), 14α­(H), 17α­(H)-sterane 20S + C_29_ 5α­(H), 14α­(H), 17α­(H)-sterane 20R); TMNr = 1,3,7-TMN/(1,3,7-TMN + 1,2,5-TMN); TeMNr = 1,3,6,7-TeMN/(1,3,6,7-TeMN + 1,2,5,6-TeMN + 1,2,3,5-TeMN); F_1_ = (3-MP + 2-MP)/(1-MP + 2-MP + 3-MP + 9-MP); F_2_ = 2-MP/(1-MP + 2-MP + 3-MP + 9-MP); GR/C_30_H = gammacerane/C_30_ hopane; DBTs/DBFs = dibenzothiophene series/dibenzofuran series; C_31_HH-22R/C_30_H = C_31_ homohopane 22R/C_30_ hopane; TTs/H = tricyclic terpane series/hopane series; St/H = regular steranes/17α­(H)-hopanes.

#### 4.5.4 Aromatic Hydrocarbons

The aromatic hydrocarbons in the K_1_ *sh* ^4^ source rock samples predominantly consist of the naphthalene series (two rings), the phenanthrene series (three rings), and the triaromatic sterane series (four rings), with the phenanthrene series exhibiting the highest abundance. Within these series, phenanthrene, methylphenanthrene, ethylphenanthrene, dimethylphenanthrene, and trimethylphenanthrene are particularly abundant. The alkylated naphthalene series shows a decreasing abundance trend in the order of trimethylnaphthalene > tetramethylnaphthalene > dimethylnaphthalene > pentamethylnaphthalene > methylnaphthalene (Figure 3d). Additionally, compounds such as biphenyl, dibenzofuran, fluorene, dibenzothiophene, benzo­[a]­anthracene, chrysene, and benzohopane were detected (Figure 3d). Geochemical parameters related to aromatic hydrocarbons are detailed in Table 6.

## 5 Discussion

### 5.1 Oil-Generating Potential of Source Rocks

According to the geochemical criteria for evaluating OM abundance in mudstones specified in the petroleum and natural gas industry standard of the People’s Republic of China (SY/T 5735–2019),^29^ most of these samples in Well LFD1 qualify as excellent source rocks, whereas a very small number of samples are classified as fair (Figure 4a). Xie et al. (2021) conducted a systematic study on the organic geochemical profiles of the K_1_ *sh* ^4^ mudstones in Well DY1,^15^ located southeast of Well LFD1 (Figure 1b). The results show that the TOC values in Well DY1 range from 1.12 to 4.71 wt %, averaging 2.73 wt %, and most samples have TOC values exceeding 2.00 wt %,^15^ generally consistent with our findings. Further examination reveals that the upper part of the K_1_ *sh* ^4^ has TOC values between 1.12 and 3.65 wt %, averaging 2.19 wt %, whereas the lower part exhibits TOC values from 1.27 to 4.71 wt %, averaging 2.85 wt %,^15^ indicating a higher enrichment of OM in the lower part. The overall increasing trend of TOC values from top to bottom of the K_1_ *sh* ^4^ in Well DY1 was also observed in the study by Xu et al.^27^ It is therefore unsurprising that the samples from Well LFD1, taken from the lower part of the K_1_ *sh* ^4^, show high TOC values ranging from 0.75 to 7.37 wt % (mean 3.98%) (Table 1). The TOC values mentioned above even exceed those of the first member of Upper Cretaceous Qingshankou Formation (K_2_ *qn* ^1^) from Well SYY1 in the Qijia Sag of the Songliao Basin, where TOC ranges from 0.64 to 4.09% with an average of 2.06%.^5^ Horizontal Well SYY1HF, guided by Well SYY1 as a pilot well, achieved a high-yield industrial shale oil production of 14.37 m^3^/day through natural flow.^9^ By analogy, it is inferred that K_1_ *sh* ^4^ in the Fuxin Basin also possesses the OM abundance basis for generating industrial quantities of shale oil.

**Figure 4:**
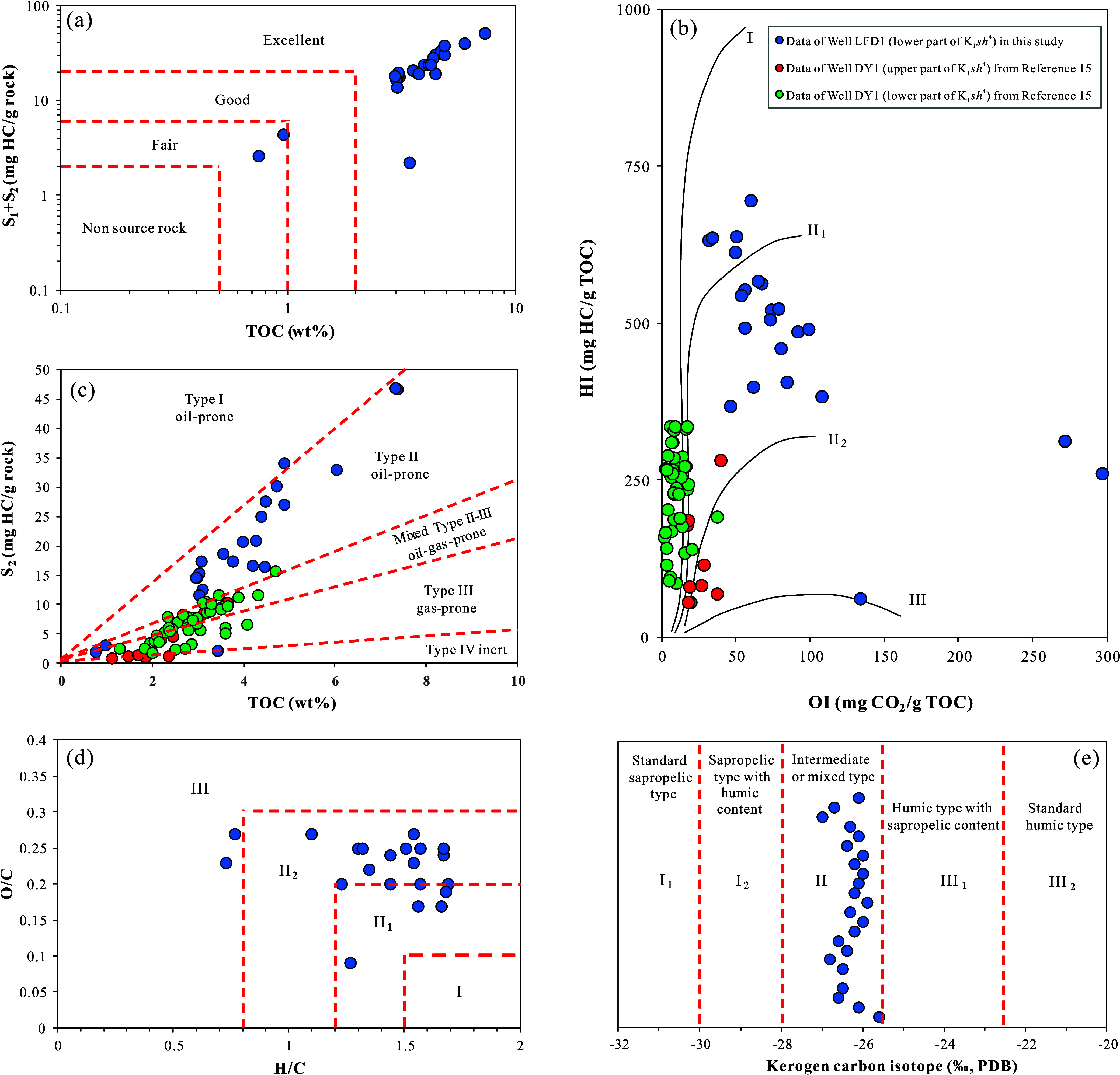
Evaluation of OM abundance and type in the K_1_ *sh* ^4^ source rock samples from Well LFD1 and Well DY1, Fuxin Basin: (a) cross-plot of S_1_ + S_2_ vs TOC; (b) cross-plot of HI vs OI; (c) cross-plot of S_2_ vs TOC; (d) cross-plot of O/C vs H/C; (e) distribution diagram of kerogen carbon isotopes. The geochemical data from Well DY1 in panels (b) and (c) were cited from Xie et al.^15^ Reproduced with permission from ref 15. Copyright 2021 Elsevier.

The IH-OI diagram reveals that the OM type of the K_1_ *sh* ^4^ source rocks in Well LFD1 is primarily Type II_1_, with Type II_2_ being the secondary type and only a very small number of samples being classified as Type III (Figure 4b). The IH values are predominantly within the range of 300–600 HC/g TOC (Table 1), and the majority of sample points are located in the oil-prone Type II region on the S_2_-TOC cross-plot (Figure 4c).^30^ From the perspective of rock pyrolysis data, these samples are inferior to the OM types in K_2_ *qn* ^1^ of Well SYY1 in the Songliao Basin (primarily Types I and II_1_),^5^ but they still belong to oil-prone source rocks. Additionally, the cross-plot of kerogen organic elemental ratios (O/C and H/C) also indicates that the OM type is mainly Type II_1_ and II_2_, with a few individual samples being Type III (Figure 4d). The kerogen carbon isotope data further show that the K_1_ *sh* ^4^ source rocks are predominantly Type II, which is indicative of an intermediate or mixed type (Figure 4e).^31^ Collectively, these results suggest that the OM type of the K_1_ *sh* ^4^ source rocks is relatively favorable, a conclusion also supported by the dominance of oil-prone sapropelinite in organic macerals (Figure 2). However, the data from Well DY1 indicate that the OM type in K_1_ *sh* ^4^ is significantly inferior to that in Well LFD1 (Figure 4b,c). This discrepancy may be attributed to two factors: (1) differences in OM sources due to the distinct sedimentary facies zones of the two wells and (2) the vertical heterogeneity of OM types within the K_1_ *sh* ^4^. Laterally, the OM types of source rocks in the Fuxin Basin show a gradual transition from Type II_1_/I to Type II_2_/III from west to east.^23^ Vertically, the lower part of the K_1_ *sh* ^4^ in Well DY1 shows markedly better OM type than the upper part (Figure 4b,c), as evidenced by the progressive depletion of carbon isotope values in the EOM of mudstones with increasing depth.^27^ The samples from the Well LFD1 belong to the lower part of the K_1_ *sh* ^4^ and are characterized by a better OM type. Additionally, this improvement in OM type aligns with the higher OM abundance observed in the lower interval.

The Ro values of the samples, which predominantly range from 0.86 to 0.94%, suggest that the K_1_ *sh* ^4^ source rocks are at a moderate maturity stage (Table 2). This conclusion is further supported by molecular geochemical parameters (Figure 5). The isomer ratios of C_31_ and C_32_ homohopanes, 22S/(22S + 22R), are 0.576–0.582 (average of 0.579) and 0.571–0.590 (average of 0.583), respectively. Given that equilibrium values range from 0.57 to 0.62, these results indicate that the samples have reached or exceeded the main oil-generating period (Figure 5a).^32^ Additionally, the regular sterane isomer ratios C_29_ 20S/(20S + 20R) and C_29_ ββ/(ββ + αα) are 0.48–0.50 (average 0.49) and 0.35–0.38 (average 0.37), respectively, confirming that the samples are at a mature stage (Figure 5b).^33^ Polycyclic aromatic hydrocarbons (PAHs) are effective indicators of the thermal evolution of source rocks, with alkyl naphthalenes and methylphenanthrenes being two of the most commonly used maturity parameters.^34–36^ For our samples, the alkyl naphthalene maturity parameters, specifically the trimethylnaphthalene and tetramethylnaphthalene ratios (TMNr and TeMNr), are 0.23–0.28 (average 0.26) and 0.43–0.46 (average 0.45), respectively. The methylphenanthrene ratios F_1_ and F_2_ are 0.432–0.438 (average 0.436) and 0.240–0.245 (average 0.243), respectively. Collectively, these parameters indicate that the samples are at a mature stage (Figure 5c–e).^37,38^ The Ro values of the K_1_ *sh* ^4^ in Well DY1 range from 0.62% to 1.11%, with an average of 0.80%,^27,39^ similar to those observed in Well LFD1. In comparison, the OM maturity of the K_1_ *sh* ^4^ mudstones in the Fuxin Basin is lower than that of K_2_ *qn* ^1^ in Well SYY1 of the Songliao Basin. The K_2_ *qn* ^1^ in Well SYY1 has reached the late mature stage (with Ro values ranging from 1.21% to 1.28%, averaging 1.26%).^5^ It indicates a relatively low degree of OM conversion to oil and gas for the K_1_ *sh* ^4^ mudstones. Nevertheless, the K_1_ *sh* ^4^ mudstones in the Fuxin Basin are currently in the oil-generation peak stage and retain a certain shale oil potential.

**Figure 5:**
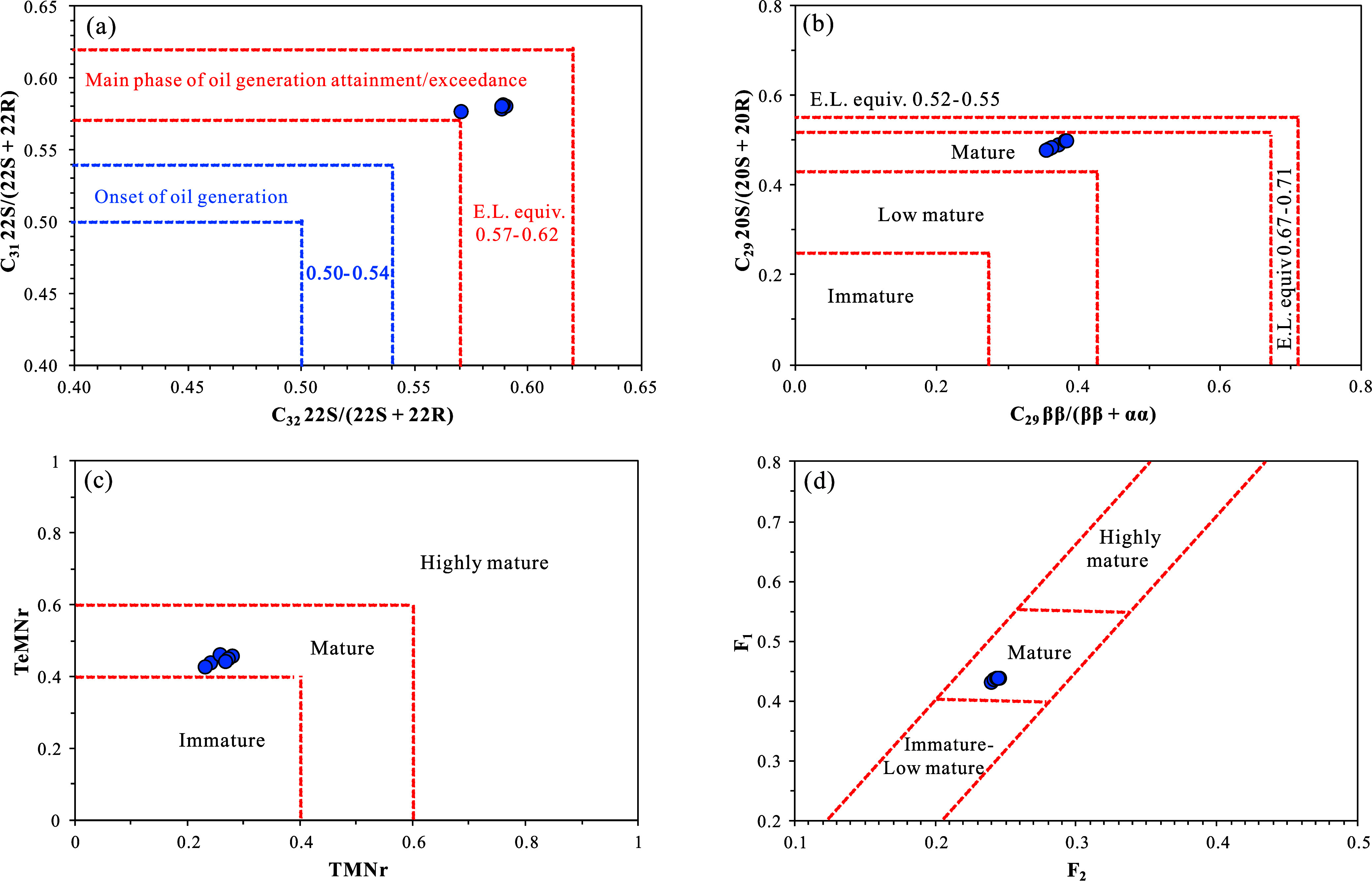
Evaluation of OM maturity in the K_1_ *sh* ^4^ source rock samples from Well LFD1, Fuxin Basin: (a) cross-plot of C_31_ 22S/(22S + 22R) vs C_32_ 22S/(22S + 22R); (b) cross-plot of C_29_ 20S/(20S + 20R) vs C_29_ ββ/(ββ + αα); (c) cross-plot of TeMNr vs TMNr; (d) cross-plot of F_1_ vs F_2_.

To sum up, the K_1_ *sh* ^4^ source rocks in Well LFD1 exhibit high OM abundance, primarily characterized by oil-prone Type II OM. These source rocks have progressed into a phase of vigorous oil generation, thereby offering a robust material foundation for the accumulation of shale oil. However, due to the heterogeneity of lacustrine sedimentation, the oil-generation potential of the K_1_ *sh* ^4^ mudstones in the Fuxin Basin may vary among different structural positions and at equivalent depths corresponding to different depositional periods. Compared to the K_2_ *qn* ^1^ in the Qijia Sag of the Songliao Basin, the K_1_ *sh* ^4^ of the Fuxin Basin exhibits less favorable OM types and maturity, but slightly higher OM abundance. Consequently, its overall shale oil material basis is weaker than that of K_2_ *qn* ^1^, yet it has reached the oil-generation threshold for shale oil enrichment.

### 5.2 Paleoclimate and Paleodepositional Environment

The interplay between paleoclimate and paleodepositional environment significantly influences the enrichment of OM and shale oil.^40,41^ On one hand, paleoclimate governs the paleobiological community and paleoproductivity, thereby controlling the biogenic type and volume of OM input, which in turn dictates the hydrocarbon generation properties and yield of the OM. On the other hand, paleoclimate directly affects the characteristics of the paleodepositional environment, such as paleoredox conditions, paleowater depth, and paleosalinity, as well as its stability, thus regulating the deposition and preservation of OM.^42–47^ Furthermore, it influences the hydrocarbon generation process and shale oil potential through the hydrocarbon-generating medium of source rocks.^48^ Additionally, the paleodepositional environment is considered the most critical factor affecting the micropore structure of low-maturity shale,^49^ implying that it determines the primary reservoir space for shale oil. In this study, trace elements and molecular geochemical data were integrated to reconstruct the paleoclimate, paleoredox conditions, and paleosalinity of the K_1_ *sh* ^4^ source rocks in the Fuxin Basin.

#### 5.2.1 Paleoclimate

The concentrations of Sr and Cu are highly sensitive to paleoclimatic changes. Sr tends to be enriched in arid and hot climates, and the Sr/Cu ratio serves as a reliable indicator for reconstructing paleoclimate.^43,46^ In the K_1_ *sh* ^4^ source rocks from Well LFD1, the Sr concentration ranges from 33.9 to 471.4 ppm, with an average of 200.8 ppm. The Sr/Cu ratio varies from 0.6 to 54.7, averaging 13.3. These data suggest that during the deposition of the K_1_ *sh* ^4^ source rocks, the climate was predominantly warm and semihumid to semiarid (Figure 6a).

**Figure 6:**
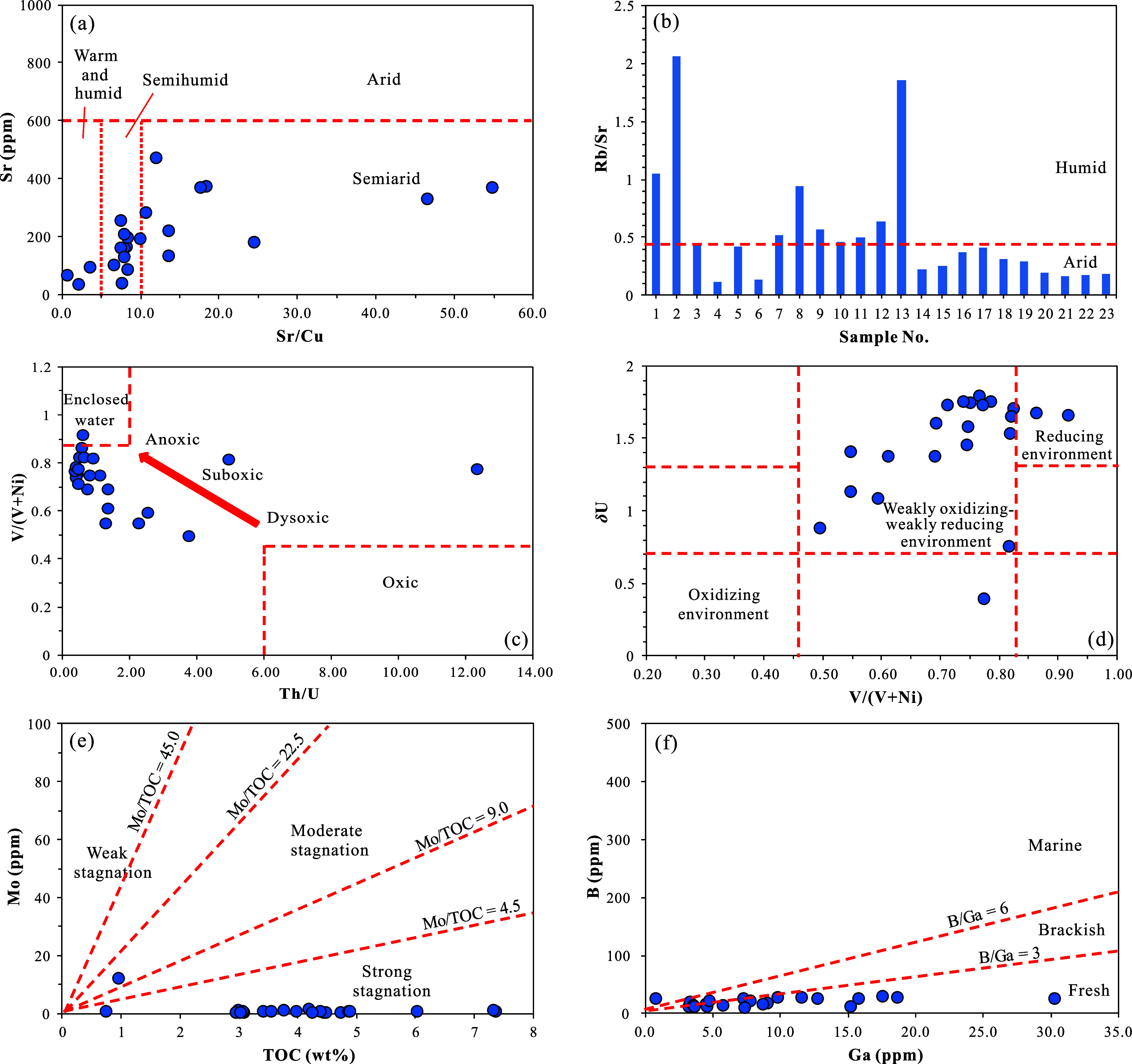
Discrimination diagrams of paleoclimate and paleodepositional environment based on trace element geochemical indicators in the K_1_ *sh* ^4^ source rock samples from Well LFD1, Fuxin Basin: (a) cross-plot of Sr concentration vs Sr/Cu; (b) distribution histogram of Rb/Sr; (c) cross-plot of V/(V + Ni) vs Th/U; (d) cross-plot of δU vs V/(V + Ni); (e) cross-plot of Mo concentration vs TOC; (f) cross-plot of B concentration vs Ga concentration.

The Rb/Sr ratio is also a commonly used indicator of paleoclimate. Under weathering, Sr is easily leached and lost, whereas Rb remains relatively stable. In humid climates, increased precipitation enhances weathering, leading to a decrease in Sr content and an increase in the Rb/Sr ratio (>0.45). In contrast, in arid climates, scarce precipitation and weak weathering result in a relatively low Rb/Sr ratio (<0.45), as rocks rich in Sr are more common.^50,51^ The Rb/Sr ratios of the samples range from 0.1 to 2.1, with an average of 0.5. Most samples cluster around 0.45, indicating that the K_1_ *sh* ^4^ was deposited under a semihumid to semiarid climate (Figure 6b).

The analysis of major elements in the source rocks of the Shahai Formation from Well DY1 indicates that the K_1_ *sh* ^3^ deposition occurred under a generally arid and cold climate, as evidenced by a low chemical index of alteration (CIA) averaging 59.89.^15^ By contrast, the bottom of the K_1_ *sh* ^4^ records a gradual shift toward a warm and humid climate, as reflected by a higher mean CIA value of 61.99; this value decreases slightly upward through the member, indicating a corresponding decline in both warmth and humidity.^15^ Additionally, Jia et al. reported a positive shift in oxygen isotope values (δ^18^O) of authigenic carbonates in the K_1_ *sh* ^4^ mudstone of the Fuxin Basin (−12 to – 9‰), along with an increase in the proportion of pteridophyte (3.5%), *Pinuspollenites* (10.3%), *Podocarpidites* (4.7%), and *Classopollis* (3.3%) in the pollen record,^52^ which may reflect a cooling trend and seasonal aridity during the K_1_ *sh* ^4^ deposition period. The samples from Well LFD1 studied here come from the lower part of the K_1_ *sh* ^4^ (Figure 1c,d); trace-element analysis therefore suggests a warm, semiarid to semihumid climate, which can be regarded as reasonable. In contrast, the K_2_ *qn* ^1^ of Well SYY1 in the Qijia Sag of the Songliao Basin was deposited in a generally humid to semihumid climate,^6^ which is more humid than that of the K_1_ *sh* ^4^ period. This environment may have been more conducive to the development of phytoplankton, thus contributing to the better OM type in the K_2_ *qn* ^1^.

#### 5.2.2 Paleoredox Conditions

The concentrations of trace elements like V, Ni, U, Th, and Mo in the strata are closely linked to the redox conditions of the depositional environment.^43,53,54^ The ratio of V to the sum of V and Ni (V/(V + Ni)) is commonly used to characterize the redox state of the water body during sedimentation. Specifically, a V/(V + Ni) ratio of <0.46 indicates an oxidizing environment, 0.46–0.83 suggests an oxygen-poor environment, and >0.83 points to an anoxic and stagnant environment.^55,56^ The Th/U ratio can also effectively reflect the redox state of the water body. A Th/U ratio of 0–2 indicates an anoxic environment, 2–6 suggests an oxygen-poor or normal environment, and >6 points to an oxidizing environment.^56^ The δU index can be used to evaluate ancient redox conditions. Generally, a δU ratio >1 indicates a reducing environment, while <1 suggests an oxidizing environment.^57^ In the samples, the V/(V + Ni) ratio ranges from 0.24 to 0.92, with an average of 0.71; the Th/U ratio ranges from 0.35 to 12.32, with an average of 1.74; and the δU ratio ranges from 0.39 to 1.79, with an average of 1.45 (Table 4). Using the V/(V + Ni)-Th/U diagram, it is determined that the K_1_ *sh* ^4^ source rocks mainly formed in an oxygen-poor depositional environment (Figure 6c). Using the δU–V/(V + Ni) diagram, it is determined that the K_1_ *sh* ^4^ source rocks mainly formed in a weakly oxidizing to weakly reducing environment (Figure 6d). The Mo abundance in relation to TOC (Mo/TOC) can be used to assess the degree of water body restriction.^58,59^ In the samples, the Mo/TOC ratio ranges from 0.05 to 12.51, with an average of 0.72, indicating an overall strongly restricted water body environment (Figure 6e), which is consistent with an oxygen-poor environment.

The sedimentary environment redox conditions of the source rocks inferred from trace element analysis are corroborated by molecular geochemical indicators of EOM. The ratio of pristane to phytane (Pr/Ph) was widely used to reflect the redox conditions of sedimentary environments. Peters et al. suggested that Pr/Ph > 3.0 indicates oxidative conditions with significant terrestrial organic matter input, Pr/Ph < 0.8 reflects typical anoxic environments, while the range of 0.8–3.0 was not recommended for specific paleodepositional environment interpretation due to confounding factors such as thermal maturity and biotic input.^32^ Therefore, the Pr/Ph ratio ranges from 0.81 to 1.08 (average 0.88, Table 5), potentially indicating a transitional environment between anoxic and oxic conditions. The Pr/*n*C_17_ vs Ph/*n*C_18_ diagram indicates that the K_1_ *sh* ^4^ source rocks were deposited in a relatively reducing water body (Figure 7a).^60^ High gammacerane content is associated with stratified, high-salinity water bodies.^33^ The ratio of gammacerane to C_30_ hopane (GR/C_30_H) is commonly used to reflect the water body salinity. The ratio of alkyl dibenzothiophenes to alkyl dibenzofurans (DBTs/DBFs) is also an effective indicator of the depositional environment.^61^ The samples exhibit very low gammacerane content with a GR/C_30_H ratio of 0.036–0.074 (average 0.058). The content of DBFs is higher than that of DBTs, resulting in a DBTs/DBFs ratio of 0.41–0.53 (average 0.47). Combining the Pr/*n*C_17_–Ph/*n*C_18_ diagram (Figure 7a),^60^ the GR/C_30_H–Pr/Ph diagram (Figure 7b),^61^ and the DBTs/DBFs-Pr/Ph diagram (Figure 7c),^62^ it is concluded that the K_1_ *sh* ^4^ source rocks were deposited in a weakly oxidizing–weakly reducing environment.

**Figure 7:**
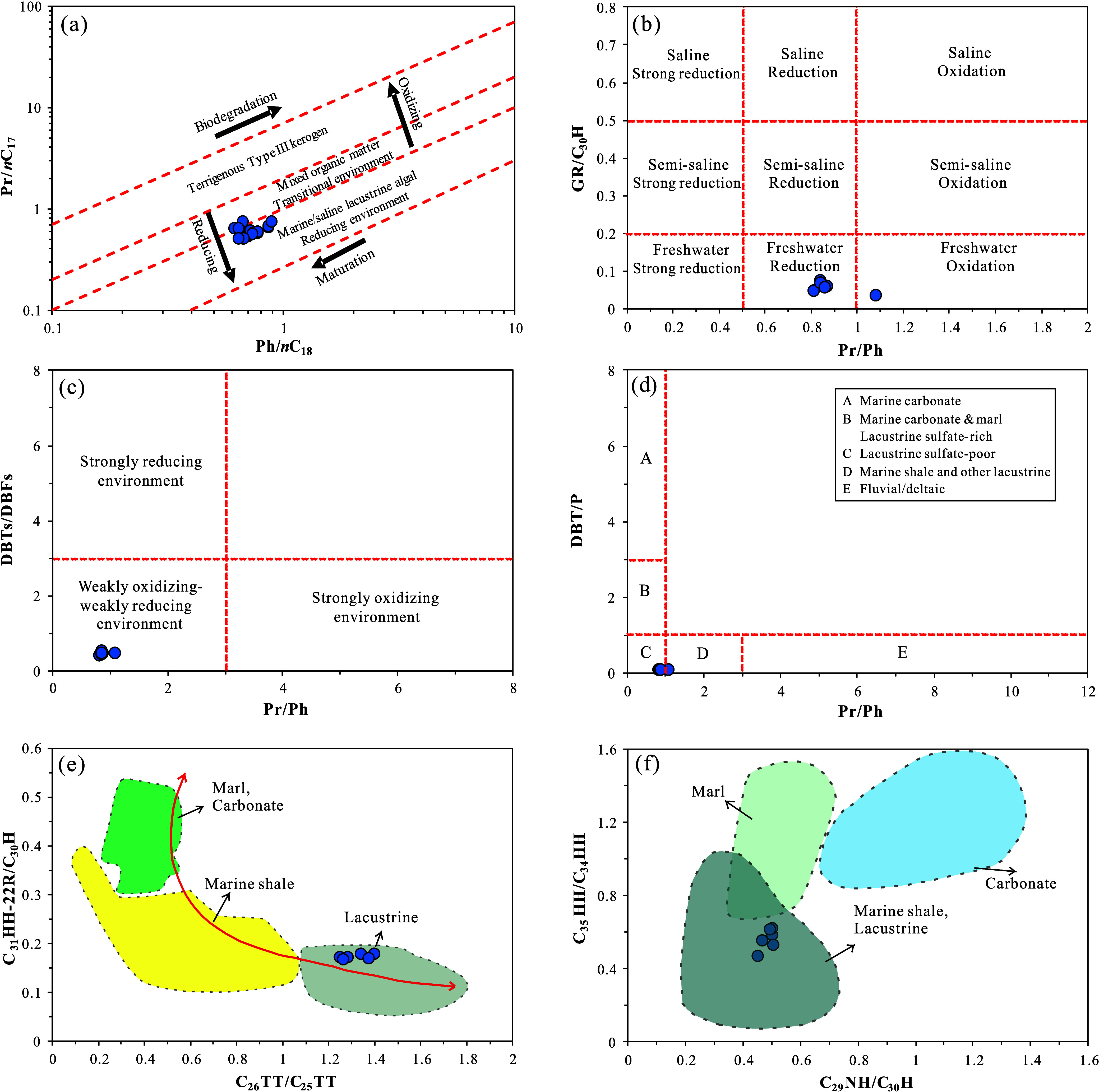
Discrimination diagrams of paleodepositional environment based on molecular geochemical indicators in the K_1_ *sh* ^4^ source rock samples from Well LFD1, Fuxin Basin: (a) cross-plot of Pr/*n*C_17_ vs Ph/*n*C_18_; (b) cross-plot of GR/C_30_H vs Pr/Ph; (c) cross-plot of DBT/P vs Pr/Ph; (d) cross-plot of DBTs/DBFs vs Pr/Ph; (e) cross-plot of C_31_HH-22R/C_30_H vs C_26_TT/C_25_TT; (f) distribution histogram of C_35_HH/C_34_HH vs C_29_NH/C_30_H.

After the transition from K_1_ *sh* ^3^ to K_1_ *sh* ^4^ in the Fuxin Basin, rapid tectonic subsidence deepened the lacustrine and expanded its surface, sustaining a persistently reducing depositional regime.^15^ Nevertheless, this reducing and oxygen-poor water column was intermittently affected by the development of gravity flows; the influx of clastic material could carry substantial amounts of oxygen, weakening the reducing conditions and causing pronounced fluctuations in relevant geochemical parameters.^15^ However, Overall, an integrated analysis of trace element and molecular geochemical indicators in Well LFD1 indicates that the K_1_ *sh* ^4^ mudstones were mainly deposited in a weakly oxidizing-weakly reducing dysoxic environment. The K_2_ *qn* ^1^ in the Qijia Sag of the Songliao Basin was primarily deposited in an anoxic stagnant deepwater to low-energy shallow-water environment.^6^ Due to the much larger lacustrine basin area compared to the Fuxin Basin, the K_2_ *qn* ^1^ was less affected by turbidity currents, resulting in more stable depositional water bodies and stronger reducing conditions than the K_1_ *sh* ^4^. This environment was more favorable for the deposition and preservation of OM.

#### 5.2.3 Paleosalinity

The B/Ga ratio in trace elements serves as a reliable geochemical indicator for assessing water salinity. Freshwater environments typically exhibit a B/Ga ratio less than 3.0, brackish water ranges from 3.0 to 6.0, and marine environments exceed 6.0.^63^ In the K_1_ *sh* ^4^ source rocks from Well LFD1, the B/Ga ratio varies from 0.8 to 32.6, with an average of 4.0. The majority of samples fall within the freshwater range, while a few plot in the brackish water zone (Figure 6f). However, the very low GR/C_30_H ratio in the K_1_ *sh* ^4^ source rocks indicates a pure freshwater depositional environment (Figure 7b, Table 6). Combined with multiple indicators discussed below, which consistently point to a freshwater–brackish water origin, the interpretation of B/Ga as a meaningful proxy appears justified. The GR/C_30_H ratio is not only related to water salinity but also to stratification in the depositional water column.^32^ Therefore, it is hypothesized that the low GR/C_30_H values in the studied samples may primarily reflect weak water stratification in the deposition period of the K_1_ *sh* ^4^ source rocks.

The ratio of dibenzothiophene to phenanthrene (DBT/P) plotted against Pr/Ph was proposed to reflect the depositional environment and lithology of source rocks.^64^ Additionally, cross-plots of the ratio of C_31_ homohopane (22R) to C_30_ hopane (C_31_HH-22R/C_30_H) vs the ratio of C_26_ tricyclic terpane to C_25_ tricyclic terpane (C_26_TT/C_25_TT) and the ratio of C_35_ homohopane to C_34_ homohopane (C_35_HH/C_34_HH) vs the ratio of C_29_ norhopane to C_30_ hopane (C_29_NH/C_30_H) can provide further insights.^32,65^ The DBT/P ratio ranges from 0.089 to 0.095, averaging at 0.093 (Table 6). Combined with Pr/Ph, this suggests that the K_1_ *sh* ^4^ source rocks are primarily sulfur-poor lacustrine mudstones or shales (Figure 7d), consistent with their relatively low DBTs content. The C_31_HH-22R/C_30_H ratio ranges from 0.169 to 0.179 (average 0.173), while the C_26_TT/C_25_TT ratio varies from 1.25 to 1.39 (average 1.32) (Table 6). These values demonstrate the characteristic ratios typical of lacustrine source rocks, namely, low C_31_HH-22R/C_30_H and high C_26_TT/C_25_TT (Figure 7e). The C_35_HH/C_34_HH ratio ranges from 0.47 to 0.62 (average 0.56), and the C_29_NH/C_30_H ratio varies from 0.45 to 0.50 (average 0.49) (Table 6), aligning with the low C_35_HH/C_34_HH and C_29_NH/C_30_H ratios characteristic of lacustrine mudstones or shales (Figure 7f). Collectively, these indicators confirm a lacustrine origin of the K_1_ *sh* ^4^ source rocks. The Pr/*n*C_17_–Pr/Ph-Ph/*n*C_18_ ternary diagram^66^ reveals that some K_1_ *sh* ^4^ source rocks have a freshwater lacustrine origin, while others exhibit brackish water lacustrine characteristics (Figure 8a). The C_19+20_TT-C_21_TT-C_23_TT ternary diagram^67^ shows most samples plotting within the transitional zone between freshwater and saline lacustrine facies (Figure 8b). Overall, the K_1_ *sh* ^4^ source rocks are predominantly of freshwater lacustrine origin with subordinate brackish water lacustrine contributions.

**Figure 8:**
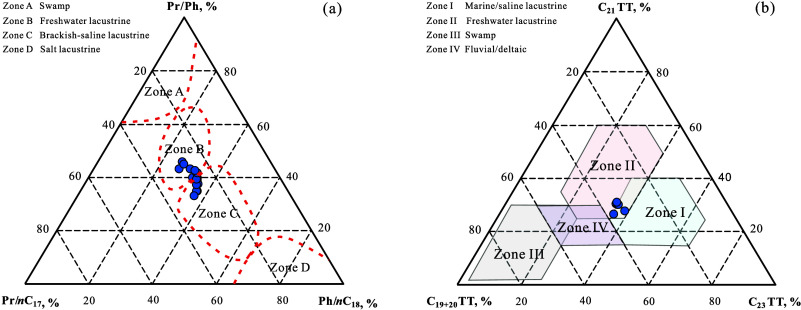
Paleosalinity ternary diagrams in the K_1_ *sh* ^4^ source rock samples from Well LFD1, Fuxin Basin, based on molecular geochemical indicators: (a) Pr/*n*C_17_–Pr/Ph–Ph/*n*C_18_ ternary diagram; (b) C_19+20_TT–C_21_TT–C_23_TT ternary diagram.

The ratio of total sulfur to total organic carbon (TS/TOC) can also serve as a salinity indicator, with values of <0.1, 0.1–0.5, and >0.5, indicating freshwater, brackish or marine, and marine, respectively.^63^ In Well DY1, the lower and upper parts of the K_1_ *sh* ^4^ exhibit average TS/TOC values of 0.12 and 0.07, respectively, indicating that the upper K_1_ *sh* ^4^ was a freshwater environment, whereas the lower K_1_ *sh* ^4^ represents a freshwater to brackish water transitional environment.^15^ The integrated geochemical evidence from Well LFD1 and Well DY1 indicates that K_1_ *sh* ^4^ in the Fuxin Basin was deposited primarily in a freshwater lacustrine environment, with subordinate brackish lacustrine facies. In terms of water salinity, the K_2_ *qn* ^1^ in the Qijia Sag of the Songliao Basin was primarily deposited in a brackish-freshwater environment.^6^ However, distinctively, the K_2_ *qn* ^1^ depositional period may have experienced marine transgression, which introduced more marine hydrogen-rich OM into the ancient lacustrine basin.^6^ This could also be one of the reasons for the superior OM type in K_2_ *qn* ^1^ compared to K_1_ *sh* ^4^ of the Fuxin Basin.

### 5.3 Biological Origin of OM

OM from diverse biological sources exhibits distinct hydrocarbon evolution pathways, oil versus gas preferences, and hydrocarbon generation potentials due to differences in chemical composition and structure,^68–70^ thereby significantly influencing shale oil enrichment. The *n*-alkanes, terpanes, and steranes present in the EOM of source rocks, with their stable molecular frameworks and characteristic distribution patterns, can effectively trace the biological origin of the original OM.^2,32,36,71^

Low- and high-carbon-number *n*-alkanes primarily originate from algae-bacteria and terrestrial higher plants, respectively.^31^ The light-to-heavy ratio (Σ*n*C_21–_/Σ*n*C_22+_) is often used to reflect the relative contribution of algae-bacteria versus terrestrial higher plants. The Σ*n*C_21–_/Σ*n*C_22+_ ratio ranges from 0.73 to 1.71, with an average of 1.26 (Table 5), indicating that the K_1_ *sh* ^4^ source rocks from Well LFD1 have mixed OM input, with the contribution of algae-bacteria generally exceeding that of terrestrial higher plants (Figure 9a). Considering the potential loss of low-carbon-number *n*-alkanes during the extraction of EOM,^72^ the actual contribution of algae-bacteria to the OM input in the source rocks should be even higher. The (*n*C_21_ + *n*C_22_)/(*n*C_28_ + *n*C_29_) ratio is relatively high, ranging from 2.02 to 5.08, with an average of 3.21 (Table 5), which also supports the above conclusion. Microscopic observation revealed abundant lamalginite components, confirming the significant contribution of aquatic algae-derived organic input.^15,23^ The TAR ratio ((*n*C_27_ + *n*C_29_ + *n*C_31_)/(*n*C_15_ + *n*C_17_ + *n*C_19_)) is commonly used to reflect the relative contribution of terrestrial plants versus aquatic organisms to the OM.^73,74^ The TAR ratio of the samples ranges from 0.20 to 0.80, with an average of 0.38 (Table 5), also indicating that the contribution of aquatic OM (algae-bacteria) is higher than that of terrestrial plants (Figure 9b). The Pr/*n*C_17_–Ph/*n*C_18_ diagram shows that the K_1_ *sh* ^4^ source rocks have mixed input or are dominated by algal OM input, with relatively less input from terrestrial higher plants (Figure 7a), consistent with the results reflected by the Σ*n*C_21–_/Σ*n*C_22+_ and TAR ratios. The *P*_aq_ ratio ((*n*C_23_ + *n*C_25_)/(*n*C_23_ + *n*C_25_ + *n*C_29_ + *n*C_31_)) can characterize the proportion of submerged/floating aquatic plants versus emergent and terrestrial plants in lacustrine sediments. *P*_aq_ < 0.1, 0.1–0.4, and 0.4–1 correspond to terrestrial plants, emergent plants, and submerged/floating plants, respectively.^75^ The *P*_aq_ ratio of the samples ranges from 0.72 to 0.85, with an average of 0.79 (Table 5), indicating that the plant OM input is dominated by submerged/floating plants, with less contribution from terrestrial/emergent plants (Figure 9c).

**Figure 9:**
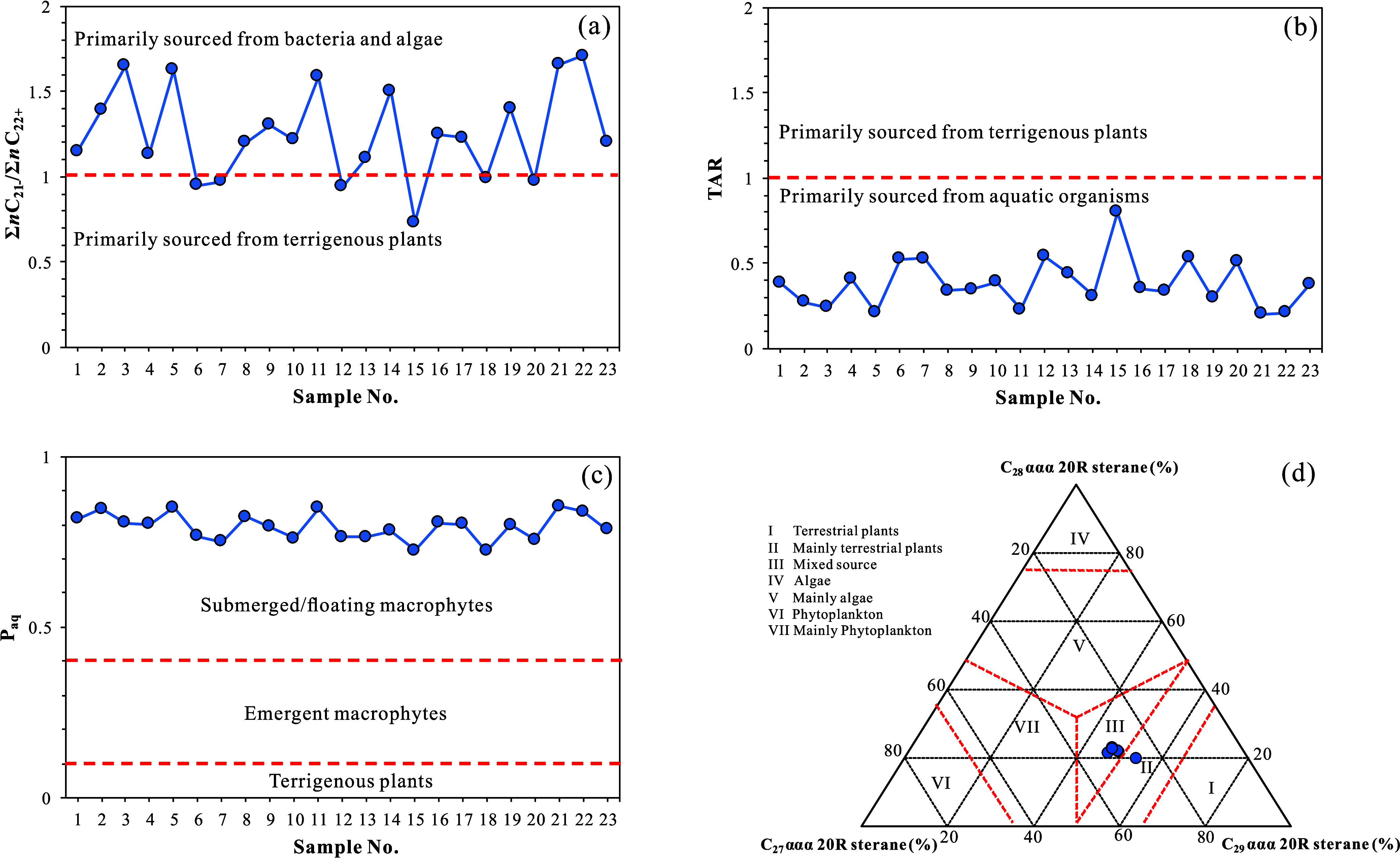
Discrimination diagrams of biological sources based on molecular geochemical indicators in the K_1_ *sh* ^4^ source rock samples from Well LFD1, Fuxin Basin: (a) distribution line chart of Σ*n*C_21–_/Σ*n*C_22+_; (b) distribution line chart of TAR; (c) distribution line chart of *P*_aq_; (d) C_27_–C_28_–C_29_ regular steranes (ααα20R) ternary diagram.

In the K_1_ *sh* ^4^ source rocks, C_19_–C_26_ tricyclic terpanes exhibit a normal distribution with C_23_ as the dominant peak, and C_24_ tetracyclic terpane is significantly more abundant than the individual isomer of C_26_ tricyclic terpanes (Figure 3b). The C_24_ tetracyclic terpane to C_26_ tricyclic terpane ratio (C_24_TeT/C_26_TT) ranges from 0.85 to 1.03, averaging 0.96 (Table 6). These characteristics closely resemble those of the Triassic lacustrine mudstones in the Kuqa Depression of the Tarim Basin, suggesting a substantial contribution from lower aquatic organisms to the OM.^76^ In general, against the backdrop of the widespread distribution of terrestrial higher plants after the Devonian, C_27_ steranes are considered to originate from planktonic algae, C_28_ steranes from specific algae such as diatoms, and C_29_ steranes from terrestrial higher plants. Therefore, the ternary diagram of C_27_–C_28_–C_29_ regular steranes (ααα20R) is commonly used to indicate the biological sources of OM in source rocks.^32,77^ Figure 9d shows that the K_1_ *sh* ^4^ source rocks received mixed inputs from both planktonic algae and terrestrial higher plants.

Steranes primarily reflect contributions from eukaryotic algae and higher plants, whereas hopane series are characteristic biomarkers of bacteria.^32^ Tricyclic terpanes, as previously mentioned, are mainly derived from aquatic organisms. On the mass chromatogram, tricyclic terpanes are significantly less abundant than hopanes (Figure 3b). The ratio of tricyclic terpanes to hopanes (TTs/H) ranges from 0.058 to 0.076, averaging 0.067 (Table 6), indicating that bacteria play a crucial role in the hydrocarbon generation of the K_1_ *sh* ^4^ source rocks. The ratio of regular steranes to 17α-hopanes (St/H) is commonly used to characterize the relative input of eukaryotic versus prokaryotic organisms.^32^ The St/H ratio of the samples ranges from 0.16 to 0.28, with an average of 0.24 (Table 6), suggesting that prokaryotic organisms dominate the biological sources of OM in the K_1_ *sh* ^4^ source rocks, or that OM has undergone significant microbial alteration.^78^ Additionally, in the *m*/*z* 191 mass chromatogram of the aromatic fraction, relatively abundant C_32_–C_35_ benzohopanes were detected (Figure 3d), further confirming the significant contribution of bacteria to the OM.

In summary, the OM in the K_1_ *sh* ^4^ source rocks is predominantly derived from algal and bacterial organisms, with prokaryotic bacteria playing a particularly crucial role in hydrocarbon generation, while the contribution from terrestrial higher plants is relatively minor. However, localized more terrestrial higher-plant-derived organic input may occur due to frequent gravity flows transporting terrigenous OM.^15^ Interestingly, in terms of the major OM sources, the K_2_ *qn* ^1^ in the Songliao Basin shows some similarity with the K_1_ *sh* ^4^ in the Fuxin Basin, particularly with prokaryotes contributing significantly.^2^ This suggests that such a combination of OM sources can be favorable for shale oil enrichment.

### 5.4 Shale Oil Enrichment and Mobility

Currently recoverable shale oil is primarily derived from potentially mobile oil within mudstones and shales. Clarifying the enrichment patterns and mobility of shale oil, as well as identifying the dominant factors controlling movable oil, are critical for achieving industrial-scale shale oil development. The following discussion primarily focuses on the enrichment characteristics and mobility of shale oil in the K_1_ *sh* ^4^ mudstones, based on geochemical indicators, with particular emphasis on the key controlling factors influencing shale oil mobility.

#### 5.4.1 Oil-Bearing Property Analysis and TOC as the Direct Factor Influencing Oil Mobility

EOM and S_1_ are the most widely used indicators for evaluating shale oil content.^79^ S_1_ values of <1.00 mg HC/g rock, 1.00–2.00 mg HC/g rock, and >2.00 mg HC/g rock correspond to low, moderate, and high oil content, respectively.^80^ S_1_ values in our samples vary from 0.12 to 6.91 mg HC/g rock, averaging 2.78 mg HC/g rock (Table 1), slightly lower than those of the K_2_ *qn* ^1^ in Well SYY1 of the Songliao Basin (1.32–7.71 mg HC/g rock; average: 3.51 mg HC/g rock).^5^ This discrepancy is closely related to the better OM type and higher maturity of K_2_ *qn* ^1^, which leads to higher residual oil content in rocks compared to the K_1_ *sh* ^4^. Most K_1_ *sh* ^4^ samples fall within the moderate to high oil content range (Figure 10a), indicating that the K_1_ *sh* ^4^ mudstones have favorable oil-bearing potential.

**Figure 10:**
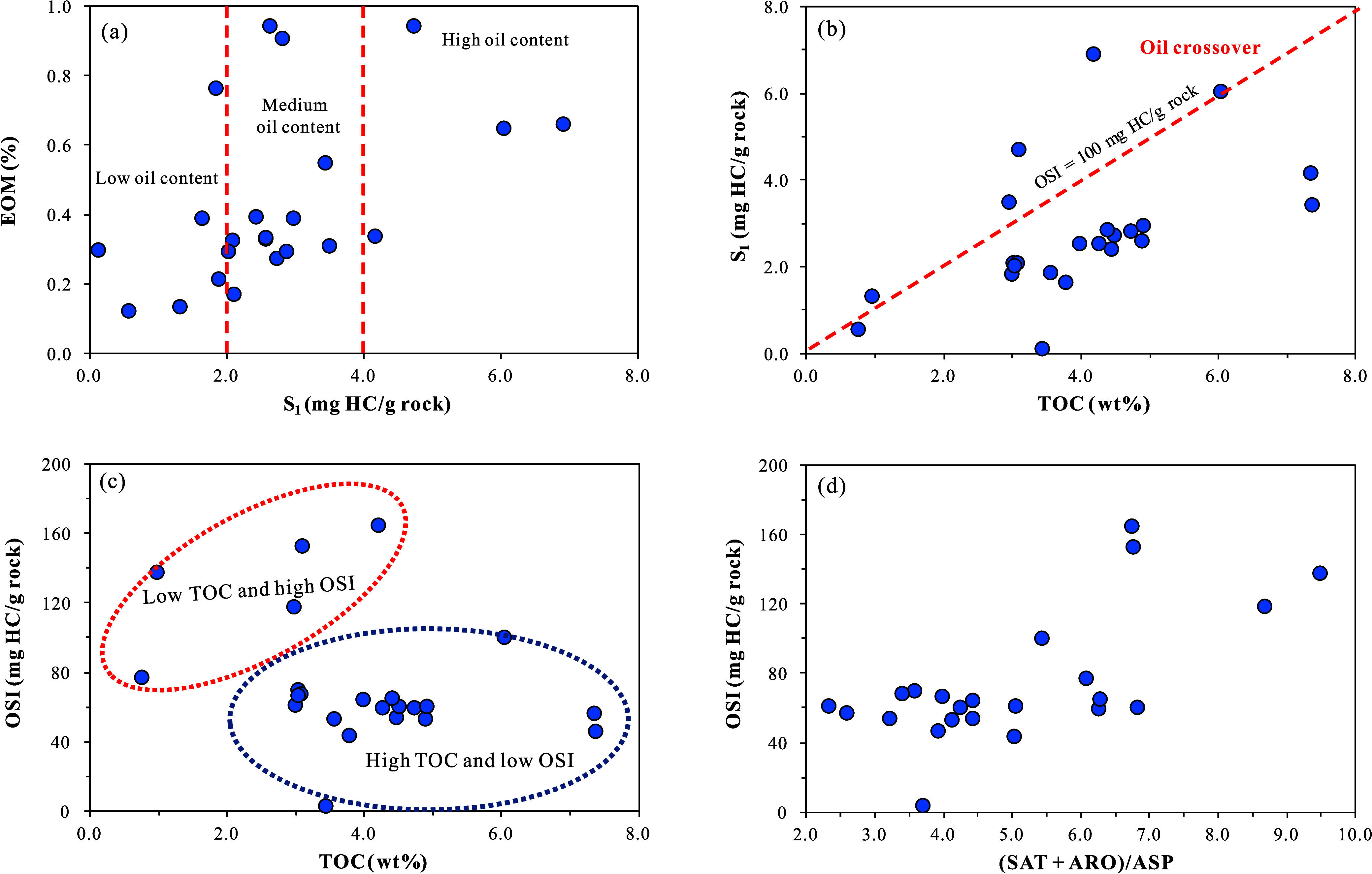
Evaluation of shale oil enrichment and distribution of the related geochemical parameters in the K_1_ *sh* ^4^ source rock samples from Well LFD1, Fuxin Basin: (a) cross-plot of EOM vs S_1_ for assessing shale oil enrichment level; (b) cross-plot of S_1_ vs TOC indicating OSI distribution; (c)­correlation diagram of OSI vs TOC; (d) correlation diagram of OSI vs (SAT + ARO)/ASP.

The oil saturation index (OSI = S_1_ × 100/TOC) is a key parameter for assessing the potential mobility of shale oil. When OSI exceeds 100 mg HC/g rock, shale oil surpasses the adsorption capacity of OM, leading to an oil crossover effect and indicating good mobility and producibility.^1^ Figure 10b shows that only a few samples exceed the OSI threshold of 100 mg HC/g rock, suggesting generally limited oil mobility. Further analysis reveals that high OSI values generally correspond to low TOC values, whereas low OSI values are typically associated with high TOC values (Figure 10c). In comparison, the OSI values of the K_2_ *qn* ^1^ in Well SYY1 of the Songliao Basin range from 63.25 to 233.16 mg HC/g rock, with an average value of 144.38 mg HC/g rock, where most samples exceed the mobile threshold of 100 mg HC/g rock.^5^ Moreover, the OSI values of the K_2_ *qn* ^1^ samples from Well SYY1 exhibit a positive correlation with TOC when TOC < 3 wt %, while it remains relatively stable when TOC > 3 wt %.^9^ These characteristics are entirely different from those of the K_1_ *sh* ^4^ samples of Well LFD1 from the Fuxin Basin. Since the S_1_ values of the K_2_ *qn* ^1^ and K_1_ *sh* ^4^ samples are not significantly different, TOC is likely the direct cause of the notably lower OSI observed in the K_1_ *sh* ^4^ samples. That is to say, there is a close relationship between shale oil mobility and the OM content. OM exhibits strong adsorption capacity for shale oil, with an adsorption capacity of approximately 100 mg oil/g TOC.^81^ Therefore, a higher TOC leads to a greater proportion of adsorbed oil and lower shale oil mobility.

Additionally, OSI is positively correlated with the ratio of saturated and aromatic hydrocarbons to asphaltenes ((SAT + ARO)/ASP) (Figure 10d), indicating that samples with high OSI values contain higher proportions of nonpolar/weakly polar hydrocarbons and lower contents of strongly polar asphaltenes. This compositional trend further confirms the high mobility of shale oil in high-OSI samples from a macroscopic component perspective. In comparison, in the EOM of K_2_ *qn* ^1^ samples from Well SYY1, Songliao Basin, saturated hydrocarbons dominate (83.62% on average), whereas aromatic hydrocarbons, nonhydrocarbons, and asphaltenes are minor (7.82, 5.94, and 2.62%, respectively).^5^ This composition gives the K_2_ *qn* ^1^ shale oil excellent mobility, consistent with its high thermal maturity (late mature stage).

#### 5.4.2 Correlations between TOC and Paleoclimate, Paleodepositional Environment, and OM Biological Sources

The above discussion has revealed the significant constraint of TOC on shale oil mobility; the following section will further analyze the correlations between TOC and paleoclimate, paleodepositional environment, as well as the biological origin of OM. Based on the analysis results mentioned above, the K_1_ *sh* ^4^ source rocks were deposited under relatively warm, semihumid to semiarid climatic conditions, in a weakly oxidizing to weakly reducing, dysoxic environment, influenced by predominantly freshwater with localized brackish deposition. The absence of significant correlations between TOC and typical paleoclimate (Sr/Cu), paleoredox (V/(V + Ni), Pr/Ph), and paleosalinity (B/Ga) indicators (Figure 11a–d) indicates that the paleoclimate and paleodepositional environment are not the controlling factors for the variable OM enrichment observed among the K_1_ *sh* ^4^ samples. However, in terms of biological input parameters, the TOC shows a positive correlation with Σ*n*C_21–_/Σ*n*C_22+_ (Figure 11e) and a negative correlation with TAR (Figure 11f), indicating that the TOC in the K_1_ *sh* ^4^ source rocks is closely related to the OM input. Specifically, a greater contribution from algae-bacteria relative to terrestrial higher plants corresponds to higher TOC content, suggesting that the sedimentary OM in the source rocks is primarily derived from algal and bacterial organisms. Therefore, a higher relative contribution of algal/bacteria-derived OM does not necessarily lead to better shale oil mobility.

**Figure 11:**
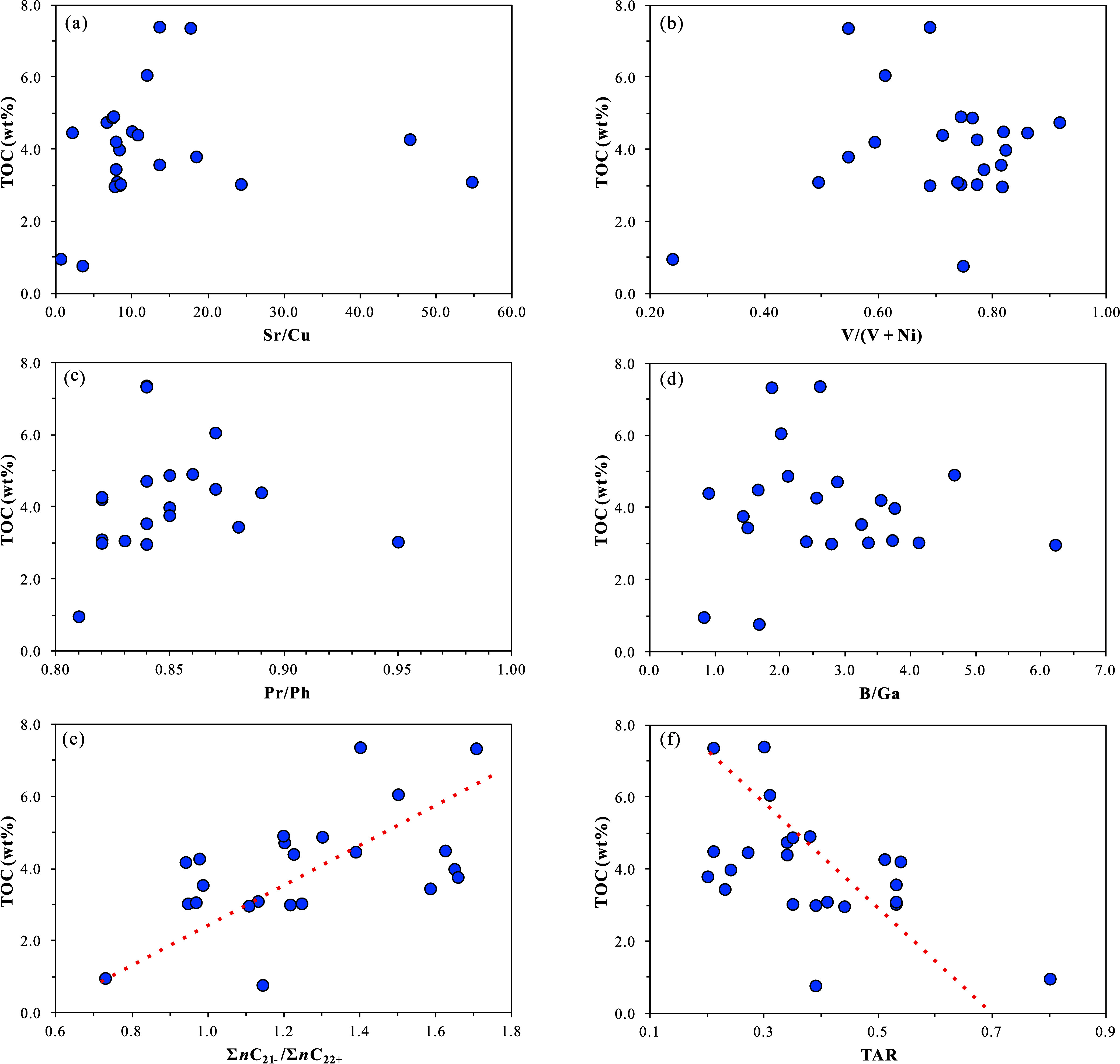
Correlation diagrams between TOC and key parameters indicative of (a) paleoclimate, (b, c) paleoredox conditions, (d) paleosalinity, and (e, f) biotic input in the K_1_ *sh* ^4^ source rock samples from Well LFD1, Fuxin Basin: (a) cross-plot of TOC vs Sr/Cu; (b) cross-plot of TOC vs V/(V + Ni); (c) cross-plot of TOC vs Pr/Ph; (d) cross-plot of TOC and B/Ga; (e) cross-plot of TOC vs Σ*n*C_21–_/Σ*n*C_22+_; (f) cross-plot of TOC and TAR.

#### 5.4.3 OM Biological Sources as the Intrinsic Control on Shale Oil Mobility

Further analysis shows that OSI exhibits a negative correlation with Σ*n*C_21–_/Σ*n*C_22+_ (Figure 12a) and a positive correlation with TAR (Figure 12b). Moreover, samples with OSI > 100 typically exhibit Σ*n*C_21–_/Σ*n*C_22+_ < ∼1.1 and TAR > ∼0.4 (Figure 12). This suggests that when the contribution from algae-bacteria is relatively low in the K_1_ *sh* ^4^ source rocks, the OM content is also lower, reducing the adsorption capacity for shale oil and thereby increasing the proportion of mobile oil. In addition, because the maturity levels of the studied samples are similar, Σ*n*C_21–_/Σ*n*C_22+_ and TAR are essentially unaffected by thermal maturity. Therefore, despite the current lack of microscopic pore structure data from shale oil reservoirs, we hypothesize that these correlations reflect an underlying control mechanism whereby the reservoir’s physical properties govern the distribution of normal alkanes with varying carbon numbers. Specifically, low Σ*n*C_21–_/Σ*n*C_22+_ and high TAR values may reflect larger pore sizes and better reservoir connectivity in the corresponding samples, thereby improving the shale oil mobility. Under such conditions, both low- and high-carbon-number *n*-alkanes can diffuse more freely,^2,82^ resulting in a more homogeneous distribution within micro- and nanoscale pores.

**Figure 12:**
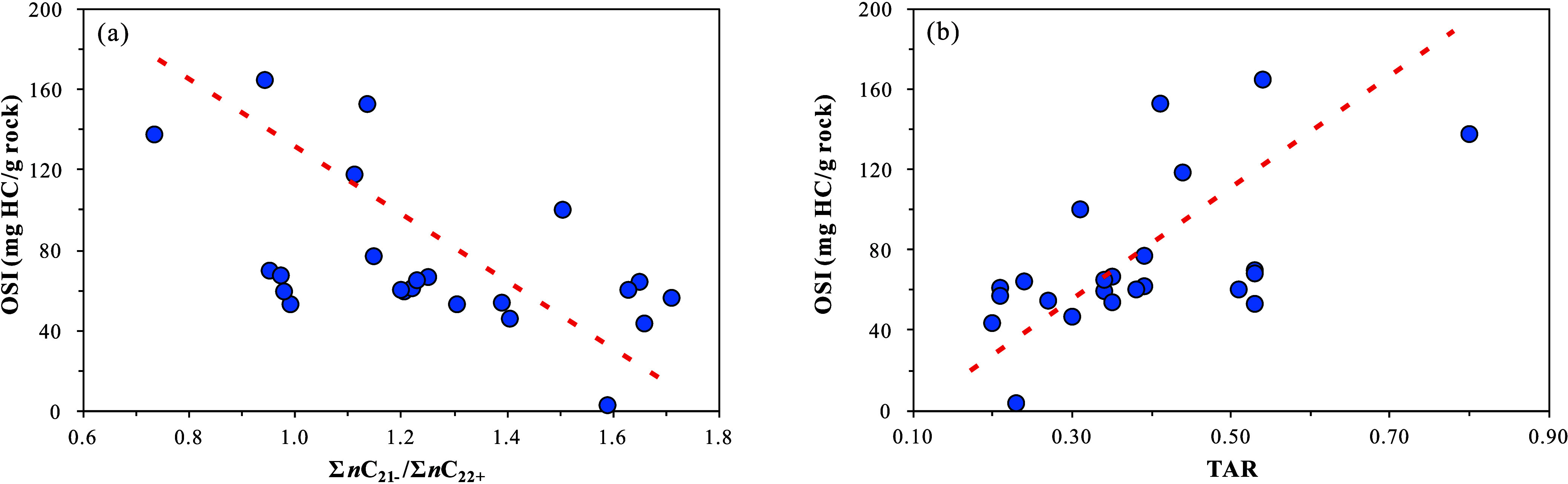
Cross-plots of (a) OSI vs Σ*n*C_21–_/Σ*n*C_22+_ and (b) OSI vs TAR in the K_1_ *sh* ^4^ source rock samples from Well LFD1, Fuxin Basin.

In micro- and nanoconfined spaces, free oil molecules migrate by diffusion, and their entry into pores is governed by diffusion energy barriers; higher barriers impede molecular passage. The magnitude of these barriers is jointly controlled by pore dimensions and molecular size: for identical pores, larger molecules face higher barriers, whereas for identical molecules, smaller pores exhibit elevated barriers (Figure 13).^83^ Consequently, the composition of free oil molecules is diffusion-energy-dependent. Mudstones and shales display pronounced heterogeneity in diffusion barriers across pore sizes: small pores selectively enrich light hydrocarbons because of high barriers, excluding heavy molecules, whereas large pores allow unrestricted diffusion of all molecular sizes, resulting in homogeneous distribution (Figure 13).^82^ In summary, it is readily apparent that, at comparable maturities, the K_1_ *sh* ^4^ samples in the Fuxin Basin characterized by low Σ*n*C_21–_/Σ*n*C_22+_ and high TAR commonly exhibit elevated OSI values and, consequently, superior shale oil mobility.

**Figure 13:**
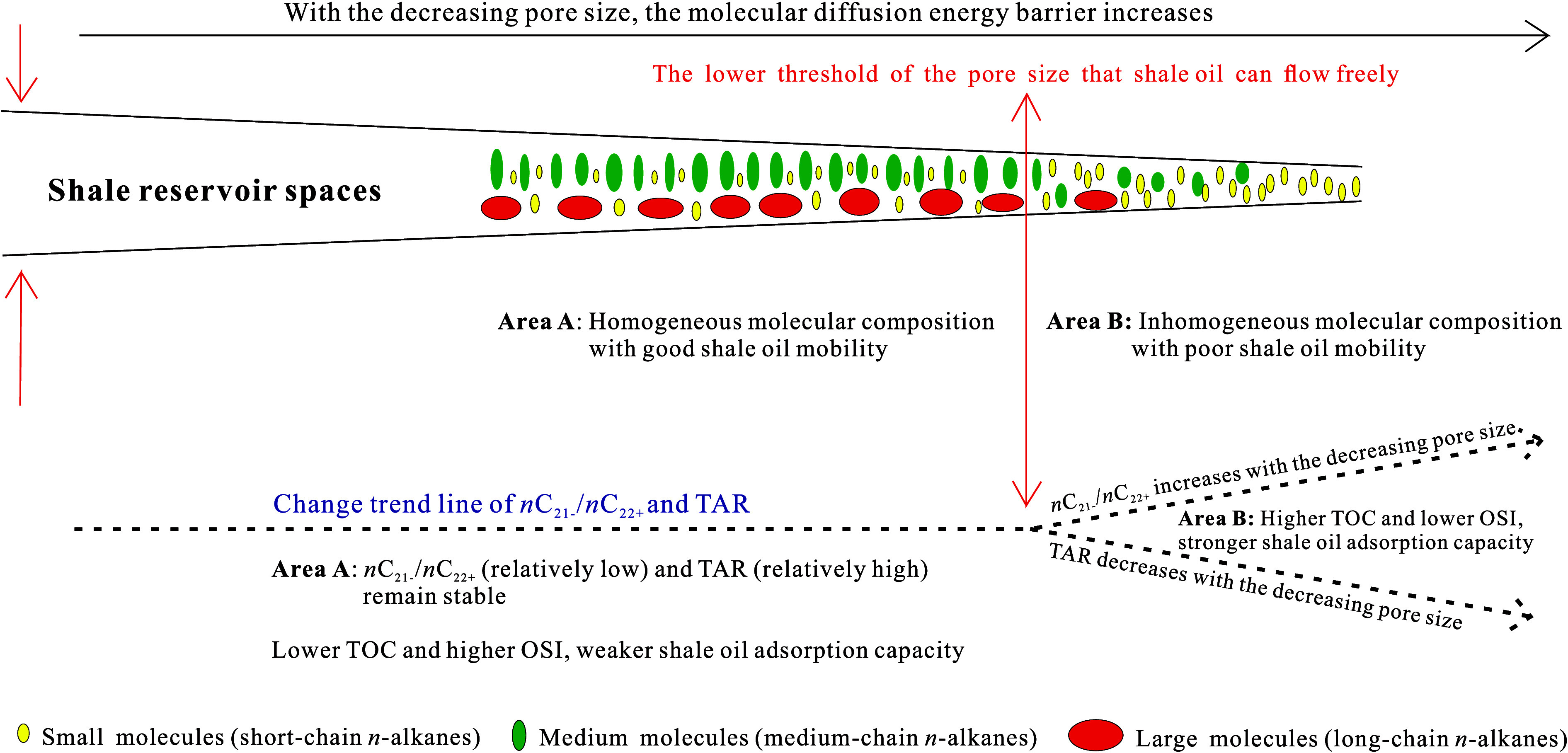
Schematic diagram illustrating the joint characterization of shale oil mobility based on reservoir pore size and geochemical parameters for the K_1_ *sh* ^4^ shale oil in Fuxin Basin. This diagram was modified from Xiao et al., 2024.^2^ Reproduced with permission from ref 2. Copyright 2024 Elsevier.

This correlation between molecular geochemical parameters (Σ*n*C_21–_/Σ*n*C_22+_) and shale reservoir pore sizes has been observed in shales from the Jiyang Depression in the Bohai Bay Basin by Jiang et al.^82^ It was demonstrated that when shale pore sizes are relatively small, light hydrocarbon compounds predominantly exist in free states, while large-molecular-weight compounds are mainly bound. Under these conditions, the free shale oil exhibit high Σ*n*C_21–_/Σ*n*C_22+_ ratios (typically >1), with S_1_ generally <3 mg/g and OSI usually <100 mg/g, indicating poor shale oil mobility. Conversely, when shale pore sizes exceed a critical threshold, long-chain hydrocarbon compounds are no longer inhibited by diffusion energy barriers and primarily exist in free states. In such cases, free oil showed low Σ*n*C_21–_/Σ*n*C_22+_ ratios (<1), with S_1_ > 3 mg/g and OSI > 100 mg/g, suggesting good mobility (Figure 13). Based on the relationship between Σ*n*C_21–_/Σ*n*C_22+_ and median pore-throat radius, the minimum flowable pore-throat radius for shale oil was estimated to be 20 nm.^82^ Coincidentally, another study found similar results. In the K_2_ *qn* ^1^ shale oil reservoirs of the Sanzhao Sag, Songliao Basin, a negative correlation was found between (*n*C_21_ + *n*C_22_)/(*n*C_28_ + *n*C_29_) and OSI.^2^ Our unpublished data further reveal close associations between geochemical indicators (e.g., (*n*C_21_ + *n*C_22_)/(*n*C_28_ + *n*C_29_) ratios) and micropore structure parameters. Therefore, while pore size data for the K_1_ *sh* ^4^ mudstones in the Fuxin Basin are currently unavailable, we hypothesize that similar correlations between normal alkane molecular fractions and pore sizes should exist based on the above findings. Further research on the mobility of shale oil in the K_1_ *sh* ^4^ mudstones of the Fuxin Basin, incorporating reservoir pore structure test data and molecular geochemical parameters, is a worthwhile endeavor for future work.

## 6 Conclusions

From 23 mudstone samples of the K_1_ *sh* ^4^ in the Fuxin Basin, we integrated inorganic–organic geochemistry with petrology to evaluate oil-generation potential, reconstruct paleoclimate and paleodepositional settings, and fingerprint the biological sources of OM. These insights constrain the enrichment and mobility of shale oil in lacustrine mudstones and yield the following key findings:

1) The K_1_ *sh* ^4^ lacustrine mudstones in the Fuxin Basin are characterized by high OM abundance and are predominantly classified as excellent source rocks. The OM is predominantly of Type II, consisting mainly of oil-prone sapropelinite as the key maceral component. These rocks have reached a stage of extensive oil generation, providing a solid material foundation for shale oil enrichment.
2) Paleoclimatic and paleosedimentary indicators reveal that the K_1_ *sh* ^4^ source rocks formed under a warm, semihumid to semiarid climate and were deposited in a weakly oxidizing to weakly reducing, dysoxic environment. These rocks chiefly accumulated in a strongly restricted, predominantly freshwater (partly brackish) lacustrine environment under relatively stable depositional conditions, which favored OM preservation and shale oil formation.
3) OM in the source rocks is predominantly derived from algae and bacteria, with prokaryotic bacteria playing a critical role in the transformation of organic material into hydrocarbons. A higher contribution from algae and bacteria sources generally corresponds to elevated organic content. In contrast, biotic input from terrestrial higher plants is relatively limited. The above biotic input provides a significantly higher proportion of oil-prone OM, thereby enhancing shale oil potential.
4) Although the lacustrine mudstones of the K_1_ *sh* ^4^ show promising oil-bearing potential, their overall oil mobility remains relatively limited. The mobility of shale oil is primarily governed by the biological origin of the OM. A lower contribution from bacteria and algae typically results in reduced OM content, which decreases the shale’s adsorption capacity and thereby enhances oil mobility. Furthermore, samples with elevated OSI values exhibit low Σ*n*C_21–_/Σ*n*C_22+_ ratios and high TAR values, possibly indicating larger and better-connected reservoir spaces that enhance shale oil mobility. Under such conditions, diffusion and fractionation of both low- and high-carbon-number *n*-alkanes are facilitated, resulting in a more uniform distribution.

In summary, the K_1_ *sh* ^4^ oil-prone mudstones can exhibit recoverable shale oil potential once they have entered or exceeded the main oil window (mid-to-late mature and high-mature stages) and preserve a biotic assemblage that limits excessive adsorption (Σ*n*C_21–_/Σ*n*C_22+_ < ∼1.1 and TAR > ∼0.4), and thus merit further exploration attention. However, it must be emphasized that on one hand, maturity significantly impacts both Σ*n*C_21–_/Σ*n*C_22+_ and TAR, leading to potential variations in their mobility thresholds under different maturity levels. On the other hand, differences in geological settings across basins, combined with distinct community development characteristics of biota and variations in sedimentary OM sources, may also result in divergent mobility thresholds for these parameters and maturity (Ro). Therefore, when applying geochemical methods to study shale oil mobility, case-specific analysis is required for individual basins. The K_1_ *sh* source rocks are widely developed in multiple small- and medium-sized basins across western Liaoning and southeastern Inner Mongolia (all within the broader Northeast China region), where K_1_ *sh* ^4^ also exhibits promising shale oil exploration potential under favorable geological conditions. Additionally, the findings of this study hold reference value for shale oil exploration in sedimentary basins with similar petroleum geological settings.

As the samples examined in this study were obtained only from specific intervals of K_1_ *sh* ^4^, the overall enrichment patterns and mobility of shale oil within the K_1_ *sh* ^4^ source rocks in the Fuxin Basin require additional investigation in future research. Moreover, this study primarily relied on molecular geochemical data to assess shale oil mobility, with limited reservoir-scale insights. To further elucidate shale oil mobility through pore size distribution and connectivity characteristics in shale reservoirs, future research should employ advanced unconventional reservoir analysis techniques.
